# Supplementary material for: Protein-polymer bioconjugates via a versatile oxygen tolerant photoinduced controlled radical polymerization approach
Source: Nat Commun. 2020 Mar 20;11:1486. doi: 10.1038/s41467-020-15259-z (PMC7083936; doi:10.1038/s41467-020-15259-z)
Supplement: Supplementary file 1 — Supplementary Information [file 41467_2020_15259_MOESM1_ESM.pdf]

Supplementary Information

Protein-Polymer Bioconjugates via a Versatile Oxygen Tolerant Photoinduced Controlled Radical Polymerization Approach

Alexis Theodorou,[1] Evelina Liarou,[2] David M. Haddleton,[2] Iren Georgia Stavrakaki,[1] Panagiotis Skordalidis,[1] Richard Whitfield,[3] Athina Anastasaki[3]* and Kelly Velonia[1]*

[1]Department of Materials Science and Technology, University of Crete, Heraklion 70013, Greece

[2]Chemistry Department, University of Warwick, Coventry, CV4 7AL, UK

[3]Department of Materials, ETH Zurich, Zurich, 8093, Switzerland

email: athina.anastasaki@mat.ethz.ch; velonia@materials.uoc.gr

Supplementary Methods

1. General Information

Materials

All materials were purchased from Sigma-Aldrich or Fischer Scientific and used as received unless otherwise specified. Deuterated solvents were obtained from Cambridge Isotope Laboratories. Bovine Serum Albumin (BSA), Glucose Oxidase from *Aspergillus niger* (GOx), beta-galactosidase from *Aspergillus oryzae* (β-gal) and ferritin were purchased from Sigma (>99%). Tris-(2-(dimethylamino)ethyl)amine (Me_6TREN) was synthesized according to the literature and stored at 4 ºC.[1] Protein SDS PAGE Molecular Weight Standards were purchased from Serva. Dialysis bags (Spectra/Por® Biotech Regenerated Cellulose Dialysis Membranes, MWCO 10 and 25 kDa) were purchased from Spectrum Labs.

Analytical Techniques

NMR Spectroscopy

NMR data were obtained for ^{1}H at 500 MHz and for ^{13}C at 125 MHz. All chemical shifts are reported in ppm (δ) relative to tetramethylsilane, referenced to the chemical shifts of residual solvent resonances (^{1}H and ^{13}C). The following abbreviations were used to explain the multiplicities: s=singlet, d=doublet, dd= doublet of doublets, t=triplet, m=multiplet.

Size Exclusion Chromatography

Aqueous size exclusion chromatography (SEC) was conducted using a Shimadzu modular system comprising a DGU-14A solvent degasser, a LC-10AD pump, a CTO-10A column oven, a SIL-10AD auto-injector, a RID-10A refractive index detector and a SPD-10A Shimadzu U.V. Vis. Spectrometer. The system was equipped with a Polymer Laboratories 30×7.8 mm 5 µm BioBasic SEC 60 guard column followed by a 300×7.8mm 5 µm BioBasic SEC 300 Polymer Laboratories column, using a mixture, of 10% MeCN in 5mM phosphate buffer pH 7.4 as the eluent at room temperature (flow rate: 0.5 mL min^{-1} or 1 mL min^{-1}). Chromatograms were acquired at 254 nm and 280 nm wavelength and were processed with the EZStart 7.3 chromatography software.

Native Polyacrylamide Gel Electrophoresis (PAGE)

Discontinuous Native PAGE (Ornstein-Davis) electrophoresis was run using a 4% stacking gel and a 10% resolving gel under standard non-denaturing conditions. Samples were dissolved in Tris buffer containing bromophenol blue and were visualized using Coomasie Brilliant Blue. Semi-quantitative digital analysis of the electrophoresis gel was performed using the using the freely available ImageJ software (https://imagej.nih.gov).[2, 3]

SDS–PAGE electrophoresis

PAGE electrophoresis was run using a 4% stacking gel and a 10% resolving gel. Samples mixed with an equal volume of electrophoresis sample buffer (125 mM Tris-HCl, pH 6.8,

5% SDS, 20% v/v glycerol, 0.004% bromophenol blue, 10% *β*-mercaptoethanol) and heated at 95 °C for 4 min prior to loading.

Matrix Assisted Laser Desorption/Ionization Time of Flight Mass Spectrometry (MALDI-ToF-MS)

MALDI-ToF-MS was conducted using a Bruker Daltonics Ultraflex II MALDI-ToF mass spectrometer equipped with a nitrogen laser delivering 10 ns laser pulses at 337 nm with positive ion ToF detection performed using an acceleration voltage of 25 kV. Solutions of CHCA (α-cyano-4-hydroxycinnamic acid) as the matrix (40 mg mL^{-1}) in acetonitrile:water 1:1, sodium trifluoroacetate as the cationization agent (1.0 mg mL^{-1}) in THF and sample (5 or 10 times dilution of a 0.2 mM product sample) were prepared. 10 µL of matrix solution was mixed with 2 µL of cationization agent solution and 10 µL of sample solution, and 0.5 µL of the mixture was applied to the target plate. Spectra were obtained linear mode in a range of 60-80 kDa.

Thermogravimetric analysis (TGA)

The thermogravimetric analyses were performed using a Perkin Elmer Pyris Diamond TG/DTA Instrument. In a typical measurement freeze-dried samples (initial weight ≤ 5 mg) set in alumina pans, were placed on a thermobalance and heated between 25 and 550°C at a heating rate of 10 °C/min under continuous nitrogen atmosphere.

Scanning Electron Microscopy

Scanning electron microscopy was performed using a ZEISS Gemini SEM - Field Emission Scanning Electron Microscope. Best results were obtained when using the InLens detector with ~3.5 mm working distance, 30 µm aperture and 2-10 kV acceleration voltage, with respect to sample tolerance. 1 µL of each sample was dissolved in 1 mL of deionized water (DI) and 7 µL aliquots were drop-casted on silicon wafer chips (5 mm x 7 mm), left to dry at room temperature and attached to aluminum specimen stubs. For the improvement of the sample imaging, gold (Au) sputter coating was applied for 15 seconds prior to imaging. SEM microscopy was also performed with a JEOL JSM 6390LV Scanning Electron Microscope operated at 15 kV.

Transmission Electron Microscopy

TEM micrographs were obtained using a JEOL JEM-2100 transmission electron microscope at an accelerating voltage of 200 kV. The samples for TEM were prepared by dropcasting 7 µL of the diluted solution (1 µL of sample dissolved in 1 mL of DI H_2O) onto lacey carbon-copper grids supplied by Agar Scientific and were left to dry at room temperature for ~2 hours. TEM experiments were also performed using a JEOL JEM-100C microscope operating at 80 kV.

Circular Dichroism (CD) Spectroscopy

Circular dichroism (CD) experiments were performed on a Jasco J-815 spectropolarimeter over the range of 180−360 nm, at 0.5 nm intervals, using a 1.0 mm path rectangular quartz cuvette supplied by Starna. Aliquots of 1 µL from the biomolecule and bioconjugate

samples were dissolved in 1 mL DI H_2O and vortexed for ~ 5 sec before being loaded in the quartz cuvette. The spectra were recorded for the samples by using a 1.0 nm bandwidth, data pitch of 0.5 nm and a response time of 1 sec. A minimum of five scans were averaged prior to the data conversion to absolute CD values. The resulting spectra were subtracted from the baseline (1 µL of 20 mM phosphate buffer in 1 mL DI H_2O).

UV Source / Other light sources
UV nail gel curing lamp (λ_{max} ~ 365 nm) with four 9 Watt bulbs was used.

Blue light: 460 nm, 50 W.

Green light: led strip 560 nm, 10.8 Wm^{-1} (2 m).

Red light: 660 nm, 100 W.

Oxygen Probe
Pocket Oxygen Meter - FireStingGO2 (from Pyro Science). The solvent-resistant oxygen probe OXSOLV measures oxygen partial pressure in most polar and nonpolar solvents. It is based on optical detection principles (REDFLASH technology) and can be used both in pure and complex organic solvents. The fiber-optic oxygen sensor tip is covered with a stainless-steel tube 1.5 mm in diameter and 150 (or 40) mm in length. The analysis of the data was conducted with the software.

2. Synthesis of the BSA-Biomacroinitiator

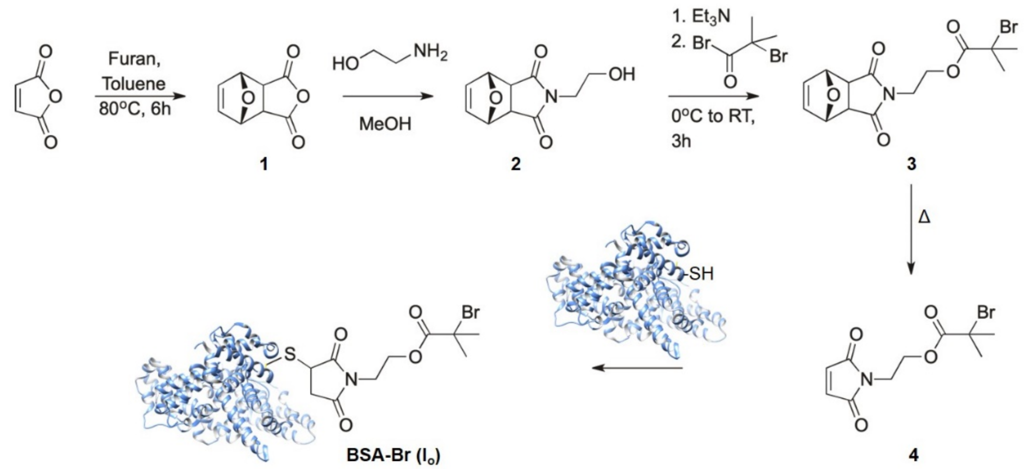

Supplementary Figure 1. Synthesis of the biomacroinitiator BSA-Br (I_o).

***N, N, N', N', N'', N''*-hexamethyl-[tris(aminoethyl)amine] Me_6TREN[1]**

18.6 mL formaldehyde 37% v/v (250.14 mmol) and 22 mL formic acid (583.1 mmol) were added to a 100 mL round bottom flask. The reaction mixture was placed in an ice bath and 3.43 mL tris(2-aminoethyl)amine (23.18 mmol) were added dropwise over the course of at least 1h, under vigorous stirring. The reaction mixture was then refluxed at 120°C for 12 h until the evolution of carbon dioxide stopped. After leaving to reach ambient temperature, the volatiles were removed by rotary evaporation under reduced pressure to afford an orange-yellow solution. The reaction mixture was placed in an ice bath and the pH was adjusted to 10 using a saturated NaOH solution (111 g NaOH in 100 mL water). The resulting oil was then extracted using 3 × 30 mL of chloroform. The combined organic layers were dried over $MgSO_4$ and the volatiles were removed by rotary evaporation under reduced pressure to yield an orange-yellow oil. The oil was distilled under reduced pressure to yield a colourless oil.

^{1}H NMR ($CDCl_3$, 500 MHz), δ (ppm): 2.62 and 2.39 (t, 12 H, (R-N-CH_2-CH_2-N-R), 2.24 (s, 18 H, (CH_3)$_2$-N-R).

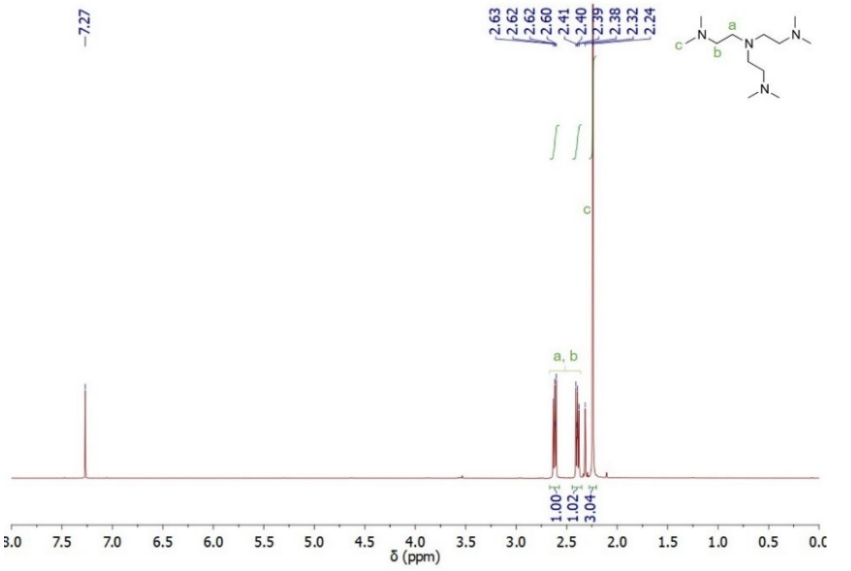

Supplementary Figure 2. ^{1}H NMR spectrum of Me_6TREN.

Synthesis of 4,10-dioxa-tricyclo[5.2.1.0^{2,6}]dec-8-ene-3,5-dione (1)[4]

Maleic anhydride (36.0 g, 367 mmol) was suspended in 180 mL of toluene and the mixture was heated to 80 °C. Furan (40.1 mL, 551 mmol) was added via syringe and the turbid solution was stirred for 6 h. The mixture was then cooled to ambient temperature under no stirring. After 1 h, the resulting white crystals were collected by filtration and washed with 2 × 30 mL of petroleum ether. 53.3 g (320 mmol, 87% yield) of the product were obtained as small white needles.

^{1}H NMR (500 MHz, $CDCl_3$, 298 K) δ = 3.18 (s, 2H, C*H*), 5.47 (t, J = 1.0 Hz, 2H, C*H*O), 6.59 (t, J = 1.0 Hz, 2H, CH_{vinyl}).
^{13}C{^{1}H} NMR (125 MHz, $CDCl_3$, 298 K) δ = 48.72 (2C, *C*H), 82.23 (2H, *C*HO), 137.00 (2C, *C*H_{vinyl}), 169.89 (2C, *C*O).

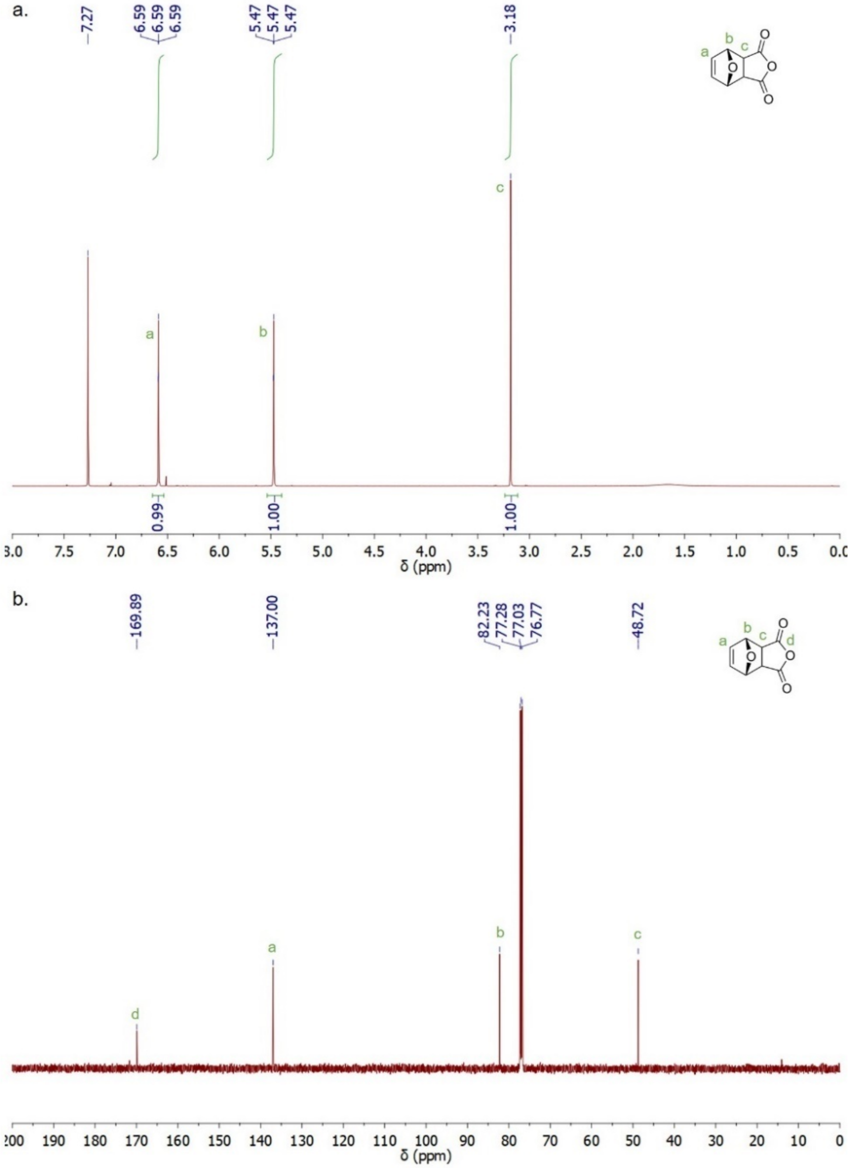

Supplementary Figure 3. ^{1}H NMR (**a**) and, ^{13}C NMR (**b**) spectra of 4,10-dioxa-tricyclo[5.2.1.0^{2,6}]dec-8-ene-3,5-dione.

Synthesis of 4-(2-hydroxyethyl)-10-oxa-4-azatricyclo[5.2.1.0^{2,6}]dec-8-ene-3,5-dione (2)[5, 6]

The anhydride **1** (2.00 g, 12.0 mmol) was suspended in MeOH (50 mL) and the mixture cooled to 0 °C. A solution of ethanolamine (0.72 mL, 12.0 mmol) in 20 mL of MeOH was added dropwise (10 min), and the resulting solution was stirred for 5 min at 0 °C, then 30 min at ambient temperature, and finally refluxed for 4 h. After cooling the mixture to ambient temperature, the solvent was removed under reduced pressure, and the white residue was dissolved in 150 mL of CH_2Cl_2 and washed with 3 × 100 mL of water. The organic layer was dried over $MgSO_4$ and filtered. Removal of the solvent under reduced pressure furnished an off-white residue that was purified by flash chromatography (CC, SiO_2, 100% ethyl acetate) to give **2** (1.04 g, 5.00 mmol, 42% yield) as a white solid.

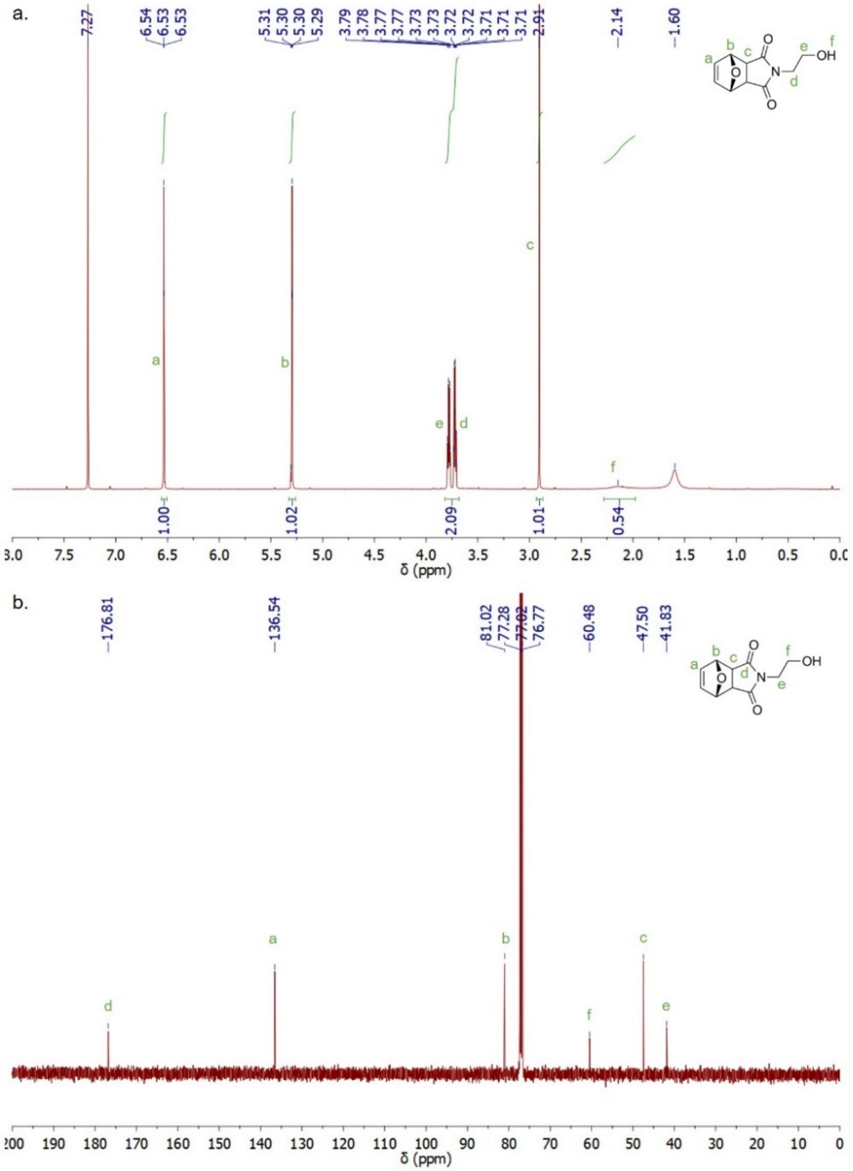

Supplementary Figure 4. ^{1}H NMR (**a**) and, ^{13}C NMR (**b**) spectra of 4-(2-hydroxyethyl)-10-oxa-4-azatricyclo[5.2.1.0^{2,6}]dec-8-ene-3,5-dione.

^1H NMR (500 MHz, $CDCl_3$, 298 K): δ = 2.10 (bs, 1H, O*H*), 2.90 (s, 2H, C*H*), 3.71-3.72 (m, 2H, NCH_2), 3.77-3.78 (m, 2H, OCH_2), 5.29 (t, J = 0.9 Hz, 2H, C*H*), 6.53 (t, J = 0.9 Hz, 2H, *C*H_{vinyl}).
$^{13}C\{^1H\}$ NMR (125 MHz, $CDCl_3$, 298 K): δ = 41.81 (1C, N*C*H_2), 47.48 (2C, *C*H), 60.46 (1C, O*C*H_2), 80.99 (2C, *C*HO), 136.52 (2C, *C*H_{vinyl}), 176.78 (2C, *C*O).

Synthesis of 2-bromo-2-methyl propionic acid 2-(3,5-dioxo-10-oxa-4-azatricyclo[5.2.1.0^{2,6}]dec-8-en-4-yl) ethyl ester (3)[5, 6]

A solution of the alcohol **2** (2.22 g, 10.6 mmol) and Et_3N (1.60 mL, 11.7 mmol) was added in 120 mL of THF to afford a slightly turbid solution which was cooled to 0 °C. A solution of 2-bromo isobutyryl bromide (1.40 mL, 11.1 mmol) in 40 mL of THF was added dropwise (over ~30 min). The white suspension was stirred for 3 h at 0 °C and subsequently at ambient temperature overnight. TLC (SiO_2, 100% ethyl acetate) revealed the complete disappearance of the starting material. The ammonium salt was filtered off and the solvent removed under reduced pressure to give a pale-yellow residue that was purified by flash chromatography (CC, SiO_2, petroleum ether/ethyl acetate 1:1). 3.54 g (9.88 mmol, 93% yield) of **3** were obtained as a white solid.

^1H NMR (500 MHz, $CDCl_3$, 298 K): δ = 1.89 (s, 6H, CH_3), 2.87 (s, 2H, C*H*), 3.81 (t, J = 5.3 Hz, 2H, NCH_2), 4.33 (t, J = 5.3 Hz, 2H, OCH_2), 5.27 (t, J = 1.0 Hz, 2H, C*H*O), 6.51 (t, J = 1.0 Hz, 2H, CH_{vinyl}).
$^{13}C\{^1H\}$ NMR (125 MHz, $CDCl_3$, 298 K): δ = 30.55 (2C, *C*H_3), 37.57 (1C, N*C*H_2), 47.46 (2C, *C*H), 55.65 (1C, *C*$(CH_3)_2$Br), 62.17 (1C, O*C*H_2), 80.82 (2C, *C*HO), 136.52 (2C, *C*H_{vinyl}),171.39 (1C, *C*O_{ester}), 175.87 (2C, *C*O_{imide}).

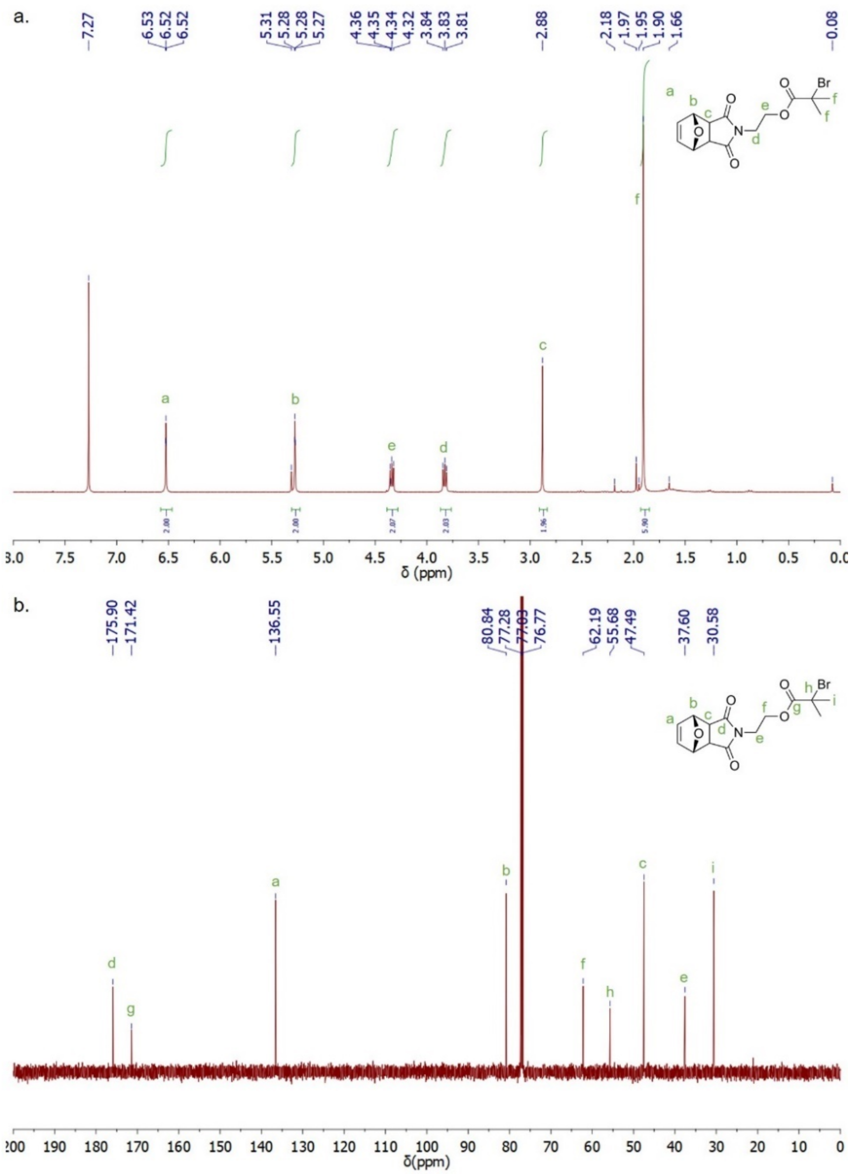

Supplementary Figure 5. ^{1}H NMR (**a**) and, ^{13}C NMR (**b**) spectra of 2-bromo-2-methyl propionic acid 2-(3,5-dioxo-10-oxa-4-azatricyclo[5.2.1.0^{2,6}]dec-8-en-4-yl) ethyl ester.

Synthesis of 2-Bromo-2-methyl-propionic acid 2-(2,5-dioxo-2,5-dihydro-pyrrol-1-yl)-ethyl ester (4)[5, 6]

0.120 g (0.335 mmol) of product **3** was suspended in toluene (5 mL), and the mixture was refluxed. The reaction was monitored by TLC (SiO_2, 100% Et_2O). After consumption of the starting material (~6 h), the solvent was removed under reduced pressure to afford **4** (0.095 g, 0.928 mmol, 98% yield) as an off-white solid.

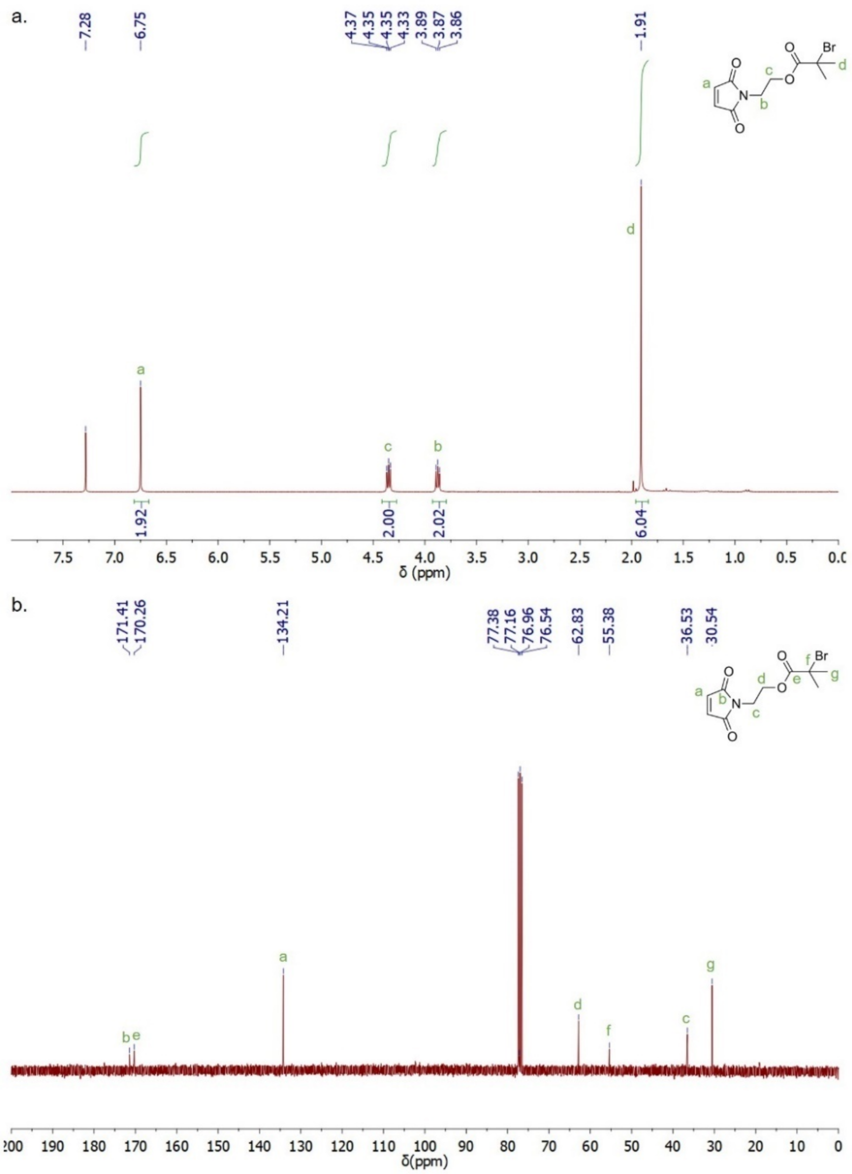

Supplementary Figure 6. ^{1}H NMR (**a**) and, ^{13}C NMR (**b**) spectra of 2-bromo-2-methyl-propionic acid 2-(2,5-dioxo-2,5-dihydro-pyrrol-1-yl)-ethyl ester.

^{1}H NMR (500 MHz, $CDCl_3$, 298 K): δ = 1.89 (s, 6H, CH_3), 3.85 (t, J = 5.3 Hz, 2H, NCH_2), 4.33 (t, J = 5.3 Hz, 2H, OCH_2), 6.73 (t, J = 1.0 Hz, 2H, CH_{vinyl}).
^{13}C{^{1}H} NMR (125 MHz, $CDCl_3$, 298 K): δ = 30.58 (2C, CH_3), 36.57 (2C, NCH_2), 55.42 (1C, $C(CH_3)_2Br$), 62.87 (OCH_2), 134.25 (2C, CH_{vinyl}), 170.30 (1C, CO_{ester}), 171.45 (2C, CO_{imide}).

Synthesis of the BSA-macroinitiator (I_o)[6]

A 150 mM solution of 2-bromo-2-methyl-propionic acid 2-(2,5-dioxo-2,5-dihydro-pyrrol-1-yl)-ethyl ester **4** in DMSO (volume accounting for 1, 10, 20 or 38 eq.) was slowly added to 9.0 mL of a 0.35 mM solution of native BSA (1 eq.) in 20 mM phosphate buffer (pH 7.4). The reaction mixture was gently shaken for 48 hours at 7°C. To eliminate the excess of **4**, the mixture was subsequently extensively dialyzed initially against 10% DMSO in 5 mM phosphate buffer pH 7.4 and then twice against 20 mM phosphate buffer pH 7.4 using regenerated cellulose dialysis membranes with a MWCO of 10 kDa. The macroinitiator was characterized by native gel electrophoresis (see Supplementary Fig. 7A) and SEC chromatography (Eluent: phosphate buffer 5 mM pH 7.4, 10 % acetonitrile, room temperature, Column: SEC-300 BioBasic, flow rate: 1.0 mL min^{-1}, see Supplementary Fig. 7B). SEC samples were prepared by dissolving 50 µL of the BSA-macroinitiator solution in 950 µL 10% MeCN in 5% phosphate buffer, pH 7.4. The BSA-macroinitiator **(BSA-Br, I_o)** solution was stored at 4 ºC. The enriched BSA-Br (Io) was additionally characterized by SDS gel electrophoresis (Supplementary Fig. 7C).

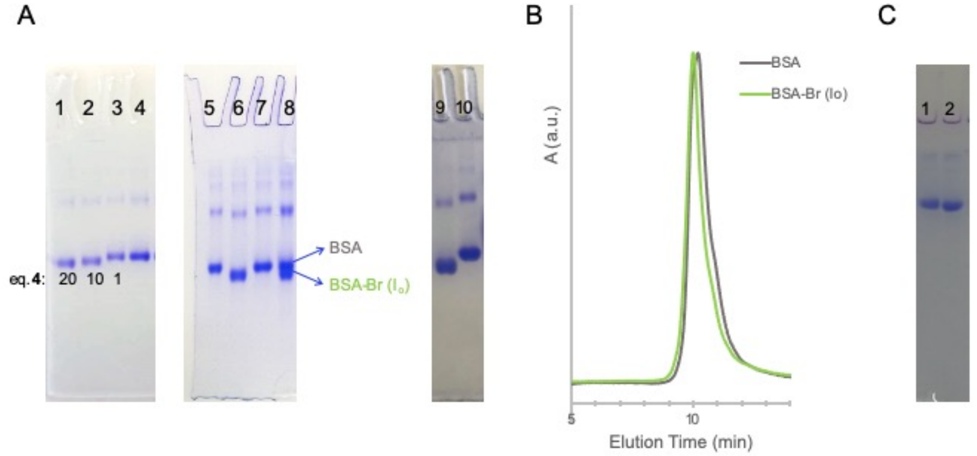

Supplementary Figure 7. Chromatographic characterization of BSA-Br macroinitiator. **(A)** Native PAGE, lane 1: Michael addition product using 20 eq. **4**, lane 2: Michael addition product using 10 eq. **4**, lane 3: Michael addition product using 1 eq. **4**, lane 4: Native BSA, lane 5: Michael addition product using 1 eq. **4**, lane 6: Michael addition product using 20 eq. **4**, lane 7: Native BSA, lane 8: Mixture of Michael addition product using 20 eq. **4** and Native BSA, lane 9: Michael addition product using 38 eq. **4**, lane 10: Native BSA. The electrophoretic gels have been cropped for clarity; **(B)** SEC chromatography, Grey trace: Native BSA, Green trace: BSA-Br; **(C)** SDS-PAGE, lane 1: BSA-Br macroinitiator (Io), lane 2: native BSA.

As shown from the electrophoretic profile of the products of the Michael addition using a varying excess of **4** over the protein, the reactions proved to be quantitative when an excess of over 20 eq. of **4** was used, while no reaction was observed with the use of 1 eq. of **4**. In our study, all reactions were performed using BSA-Br (I_o) synthesized using a 20 or ~40 fold excess of **4** during the Michael addition.

Characterization with SDS PAGE revealed the expected faster migration of denatured BSA as compared to the BSA-Br macroinitiator and a lower difference between the electrophoretic mobility of BSA and BSA macroinitiator than Native PAGE. For this

reason, native PAGE was used to monitor the vast majority of bioconjugation and grafting reactions (Supplementary Fig. 7C).

3. Oxygen Tolerant, Photoinduced Grafting of Styrene from the BSA-Biomacroinitiator

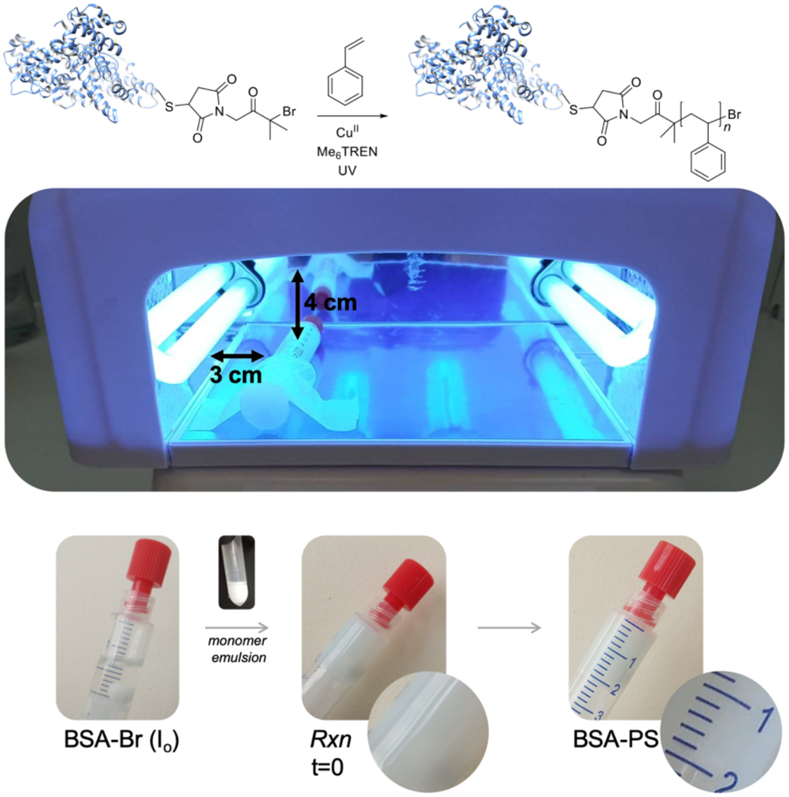

Supplementary Figure 8. Oxygen tolerant, photoinduced grafting of styrene from BSA-Br. Reaction scheme (top), experimental setup (*middle*) and reaction syringe (*bottom*) prior and after the completion of the reaction.

General Procedure of Oxygen Tolerant Photoinduced ATRP grafting of styrene from BSA-Br ([styrene]/[BSA-Br]/[Cu^{II}]/[Me_6TREN] = 5000/1/1.5/12, Table 1, entry 2)
A solution consisting of styrene (0.25 mL, 2.185 mmol, 5000 equiv.) and nanopure water (0.3 mL) was sonicated for 30 sec to form an emulsion. Me_6TREN (14 µL, 52.44 × 10^{-3} mmol, 120 equiv.) was added to 1 mL of a 1.5 mg mL^{-1} solution of $CuBr_2$ (6.55 mmol, 15 equiv.) in nanopure water to form a light blue colored solution due to the immediate copper-ligand complex formation. 100 µL of the $CuBr_2$/Me_6TREN solution (12 equiv. Me_6TREN and 1.5 $CuBr_2$ equiv.) were added to the monomer emulsion and immediately transferred to a 5 mL syringe equipped with a stirring bar, containing a 0.32 mM solution of the BSA-macroinitiator (**BSA-Br**) in 20 mM phosphate buffer, pH 7.4 (1.25 mL, 0.437×10^{-3} mmol). Headspace was eliminated to avoid the presence of undissolved oxygen and the reaction syringe was hermitically capped and placed under the UV or other light sources for specified amounts of time. Dialysis or removal of the monomer under reduced pressure preceded chromatography in all aliquots withdrawn from the reaction vessel for SEC and PAGE analysis.

A relatively high ligand loading feed of the ligand was utilized to ensure an excess of the tertiary amine would be available to mediate the reduction of the copper complex. To this

end, the pH change in the reaction mixture (pH 7.66) caused by the addition of the complex Cu^{II}/Me_6TREN was not significant to protein stability.

General Procedure of Oxygen Tolerant Photoinduced grafting of styrene from BSA-Br with DMSO as cosolvent ([styrene]/[BSA-Br]/[Cu^{II}]/[Me_6TREN] = 5000/1/1.5/12, Table 1, entry 1)

A solution consisting of styrene (0.25 mL, 2.185 mmol, 5000 equiv.), DMSO (0.2 mL) and nanopure water (0.1 mL) was sonicated for 30 sec to form an emulsion. Me_6TREN (14 µL, 52.44×10^{-3} mmol, 120 equiv.) was added to 1 mL of a 1.5 mg mL^{-1} solution of $CuBr_2$ (6.55 mmol, 15 equiv.) in nanopure water to form a light blue colored solution due to the immediate copper-ligand complex formation. 100 µL of the $CuBr_2$/Me_6TREN solution (12 equiv. Me_6TREN and 1.5 $CuBr_2$ equiv.) were added to the monomer emulsion and immediately transferred to a 5 mL syringe equipped with a stirring bar, containing a 0.32 mM solution of the BSA-macroinitiator (**BSA-Br, I_o**) in 20 mM phosphate buffer, pH 7.4 (1.25 mL, 0.437×10^{-3} mmol). Headspace was eliminated to avoid the presence of undissolved oxygen and the reaction syringe was hermitically capped and placed under the UV or other light sources for specified amounts of time. Dialysis or removal of the monomer under reduced pressure preceded chromatography in all aliquots withdrawn from the reaction vessel for SEC and PAGE analysis.

4. Optimization Studies

The general procedures of oxygen tolerant photoinduced polymerization described above were followed in all studies aiming to optimize the reaction (Supplementary Table 1). The total volume of the reaction mixtures was kept constant via the addition of the appropriate amount of nanopure water at each variation. The relevant quantities are included in Supplementary Table 1.

Supplementary Table 1. Optimization of Oxygen Tolerant Photoinduced ATRP grafting of styrene from BSA-Br

Entry	$M_n/I_o/Cu^{II}/L$	Styrene (mmole)	Cu^{II} (mmole)	Cu^{II} (ppm/mM)	Me_6TREN (mmole)	Rxn time (min)	Light Source (nm)	BSA-Br (I_o) Consumption
1	5000/1/0/0	2.185	-	-	-	480	365	no reaction
2	5000/0/1.5/12	2.185	0.655×10^{-3}	22/0.34	5.244×10^{-3}	480	356	no reaction
3	5000/1/1.5/12	2.185	0.655×10^{-3}	22/0.34	5.244×10^{-3}	480	dark	no reaction
4	5000/1/1.5/12	2.185	0.655×10^{-3}	22/0.34	5.244×10^{-3}	180	365	quantitative
5	5000/1/1.5/12	1.093	0.327×10^{-3}	11/0.17	2.622×10^{-3}	180	365	quantitative
6	5000/1/0.4/2	2.185	0.175×10^{-3}	6/0.09	0.874×10^{-3}	480	365	quantitative
7	5000/1/0.1/6	2.185	0.44×10^{-4}	1.5/0.02	2.622×10^{-3}	480	365	no or partial reaction
8	2000/1/0.4/2	0.874	0.175×10^{-3}	6/0.09	0.874×10^{-3}	480	365	quantitative
9	2000/1/1.5/12	0.874	0.655×10^{-3}	22/0.34	5.244×10^{-3}	300	365	quantitative
10	500/1/1.5/12	0,218	0.655×10^{-3}	22/0.34	5.244×10^{-3}	480	365	quantitative
11	50/1/1.5/12	0,022	0.655×10^{-3}	22/0.34	5.244×10^{-3}	480	365	no or partial reaction
12	5000/1/1.5/12	2.185	0.655×10^{-3}	22/0.34	5.244×10^{-3}	180	sunlight	quantitative
13	5000/1/1.5/12	2.185	0.655×10^{-3}	22/0.34	5.244×10^{-3}	180	blue[a]	quantitative
14	5000/1/1.5/12	2.185	0.655×10^{-3}	22/0.34	5.244×10^{-3}	180	green[b]	no reaction
15	5000/1/1.5/12	2.185	0.655×10^{-3}	22/0.34	5.244×10^{-3}	180	red[c]	no reaction
16	2000/1/1.5/12 *with headspace*	0.874	0.655×10^{-3}	22/0.34	5.244×10^{-3}	300	365	no reaction
17	2000/1/1.5/1.5	0.874	0.655×10^{-3}	22/0.34	0.655×10^{-3}	300	365	no reaction
18	2000/1/1.5/1.5	0.874	0.655×10^{-3}	22/0.34	5.244×10^{-3}	300	365 (9 Watt)	traces BSA-Br

M_n: monomer (styrene), I_o: initiator (BSA-Br), L: ligand (Me_6TREN), OC: organic content, Rxn time: reaction time.

All reactions were performed using 0.437×10^{-3} mmole BSA-Br (I_o), except the blank reaction (described in Entry 2) where no initiator was added, and reaction 5 (where 0.2185×10^{-3} mmol of BSA-Br was used).

[a] Blue light: 460 nm, 50 W.

[b] Green light: led strip 560 nm, 10.8 Wm^{-1} (2 m).

[c] Red light: 660 nm, 100 W.

BSA-PS, feed ratio [styrene]/[BSA-Br]/[Cu^{II}]/[Me_6TREN] = 5000/1/1.5/12 (Supplementary Table 1, Entries 4-7)

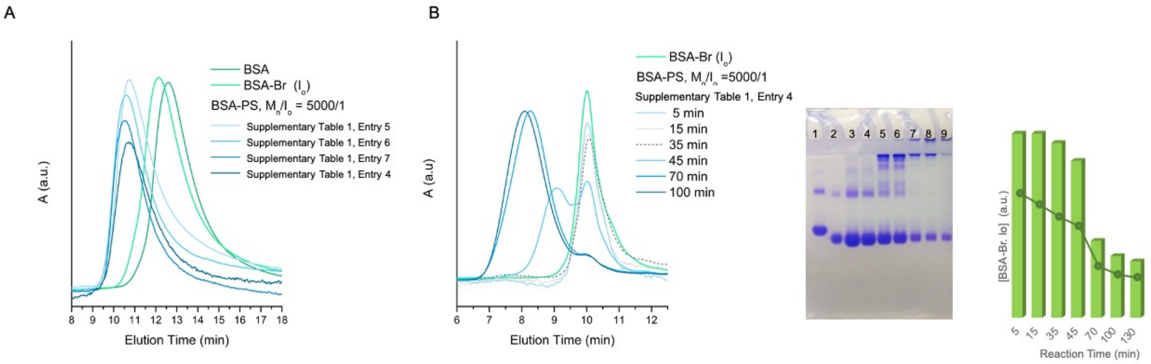

Supplementary Figure 9. BSA-PS produced via oxygen tolerant photoinduced grafting from BSA-Br using feed ratio [styrene]/[BSA-Br]/[Cu^{II}]/[Me_6TREN] = 5000/1/1.5/12 (Supplementary Table 1, entry 4). **A)** SEC traces; **B)** Time course of [styrene]/[BSA-Br]/[CuII]/[Me_6TREN] = 5000/1/1.5/12 reaction (Supplementary Table 1, Entry 5). *Left*: SEC traces; *Middle*: Native PAGE lane 1: native BSA, lane 2: BSA-Br (I_o), lane 3: 5min, lane 4: 15 min, lane 5: 35 min, lane 6: 45 min, lane 7: 70 min, lane 8: 100 min, lane 9: 130 min. The electrophoretic gel has been cropped for clarity. *Right*: Semiquantitative analysis plot of BSA-Br (I_o) consumption during the course of the reaction.

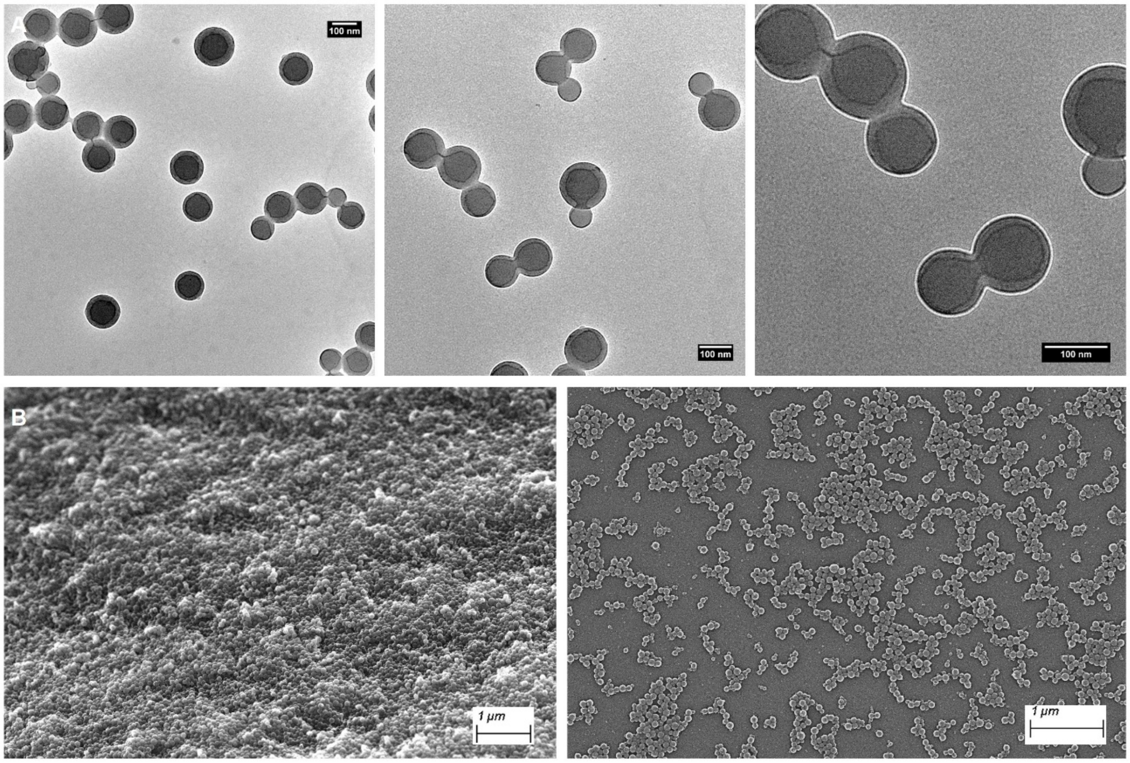

Supplementary Figure 10. Morphological characterization of BSA-PS. **A)** TEM and **B)** SEM micrographs of BSA-PS synthesized using molar feed ratio [Mn]/[I_o]=5000/1 (Supplementary Table 1, Entry 4).

Protein Digestion, Isolation of Polystyrene (Supplementary Table 1, Entry 4)
2.5 mL of the BSA-PS (0.23 mM) were dialyzed against nanopure water to remove buffer and consequently placed in a round bottom flask. 2.5 mL of 40% NaOH were added and the resulting mixture was heated for 72 hours at 95 °C. After cooling to 0 °C, the suspension was acidified to a pH between 1 and 3 using aq. HCl 6M. The resulting aqueous phase was extracted with $CHCl_3$ (2×20 mL) and the combined organic layers were washed with brine (15 mL). The organic phase was dried over $MgSO_4$ and the solvent was removed in vacuo. The polymer was precipitated twice with methanol prior to drying and analyzing with ^{1}H-NMR spectroscopy (Supplementary Fig. 11A).

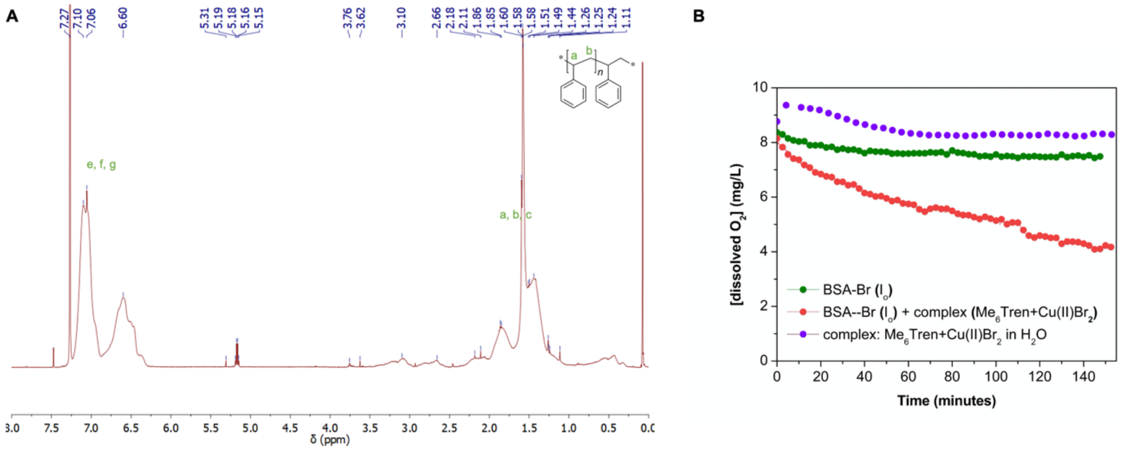

Supplementary Figure 11. Structural characterization of polystyrene and oxygen consumption study. **A)** ^{1}H NMR spectrum of polystyrene digested from BSA-PS *Giant Amphiphiles*; **B)** Line graphs illustrating the effect of the initiator BSA-Br and the complex Cu^{II}/Me_6TREN on the evolution of the dissolved oxygen concentration during polymerization.

Oxygen Probe Measurements for during the polymerization of styrene at [styrene]/[BSA-Br]/[Cu^{II}]/[Me$_6$TREN] = 5000/1/1.5/12
The oxygen consumption behaviour of the polymerization system was examined through in situ online monitoring of the dissolved O_2 concentration over time for the photoinduced grafting of styrene from BSA-Br ([styrene]/[BSA-Br]/[CuII]/[Me$_6$TREN] = 5000/1/1.5/12), and each component that comprises the polymerization was also investigated (Supplementary Fig. 11B). The heterogenous nature of the polymerization mixture (*i.e.* emulsion) did not allow for probe measurements in the presence of monomer.

Light Source (BSA-PS, Supplementary Table 1, Entries 12-15, 18)

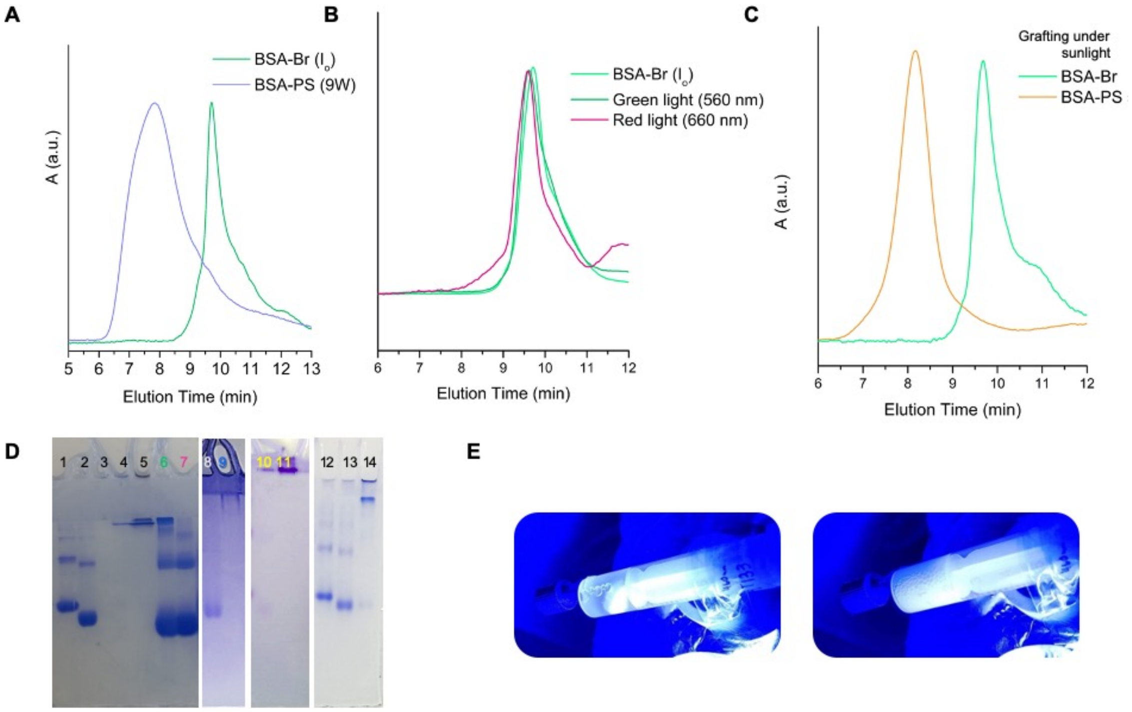

Supplementary Figure 12. Chromatographic characterization of BSA-PS produced via oxygen tolerant photoinduced grafting from BSA-Br using different light sources (Supplementary Table 1, Entries 12-15 and 18). **A)** SEC trace of reaction performed using a single UV lamp (broad band ~365 nm, 9 Watt); **B)** SEC trace of reactions performed using green or red light did not afford any products; **C)** SEC trace of reaction performed under sunlight; **D)** PAGE lane 1: native BSA, lane 2: BSA-Br, lane 6: reaction mixture after exposure to green light, lane 7: reaction mixture after exposure to red light, lane 9: BSA-PS produced via blue light photoinduced grafting of styrene, lanes 10 and 11: BSA-PS produced via sunlight photoinduced grafting of styrene, lane 12: native BSA, lane 13: BSA-Br, lane 14: BSA-PS under a 9 Watt UV (365 nm) lamp. The electrophoretic gels have been cropped for clarity; **E)** Photographs of the reaction syringe under blue light at the beginning (*left*) and after 2 hours or exposure (*right*).

BSA-PS, feed ratio [styrene]/[BSA-Br]/[Cu^{II}]/[Me$_6$TREN] = 2000/1/0.4/2 and 2000/1/1.5/12 (Supplementary Table 1, Entries 8, 9)

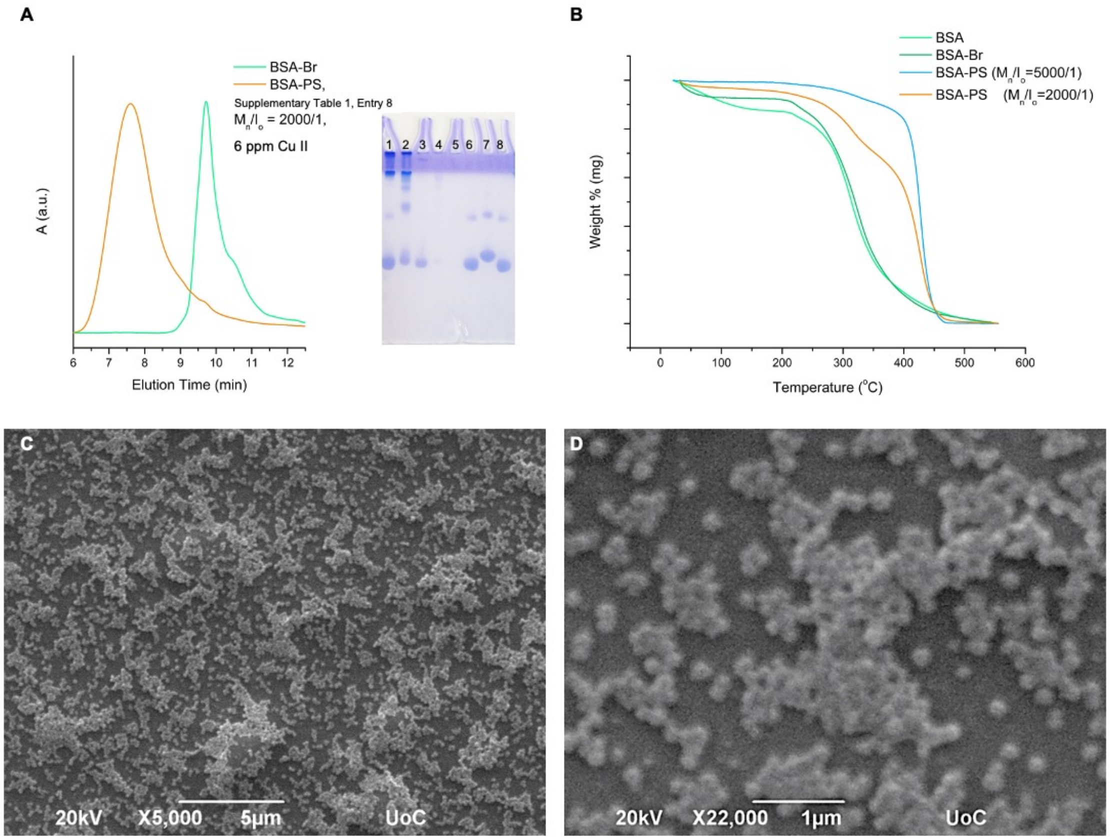

Supplementary Figure 13. Chromatographic, thermogravimetric and morphological characterization of BSA-PS produced via oxygen tolerant photoinduced grafting from BSA-Br using feed ratio [styrene]/[BSA-Br]/[Cu^{II}]/[Me$_6$TREN] = 2000/1/0.4/2 and 2000/1/1.5/12 (Supplementary Table 1, Entries 8 and 9 respectively). **A)** *left:* SEC trace; *right:* PAGE lane 1: BSA-PS, 6 ppm Cu(II) (Supplementary Table 1, Entry 8), 4h, lane 2: BSA-PS, 6 ppm Cu(II) (Supplementary Table 1, Entry 8), 8h, lane 6: BSA-Br (I_o), lane 7: native BSA. The electrophoretic gel has been cropped for clarity. **B)** Thermograms of BSA, BSA-Br and BSA-PS conjugates (Supplementary Table 1, Entry 4 and Entry 9); **C)** and **D)** SEM micrographs of BSA-PS (Supplementary Table 1, Entry 8).

BSA-PS, feed ratio [styrene]/[BSA-Br]/[Cu^{II}]/[Me$_6$TREN] = 500/1/1.5/12 and 50/1/1.5/12 (Supplementary Table 1, Entries 10 and 11)

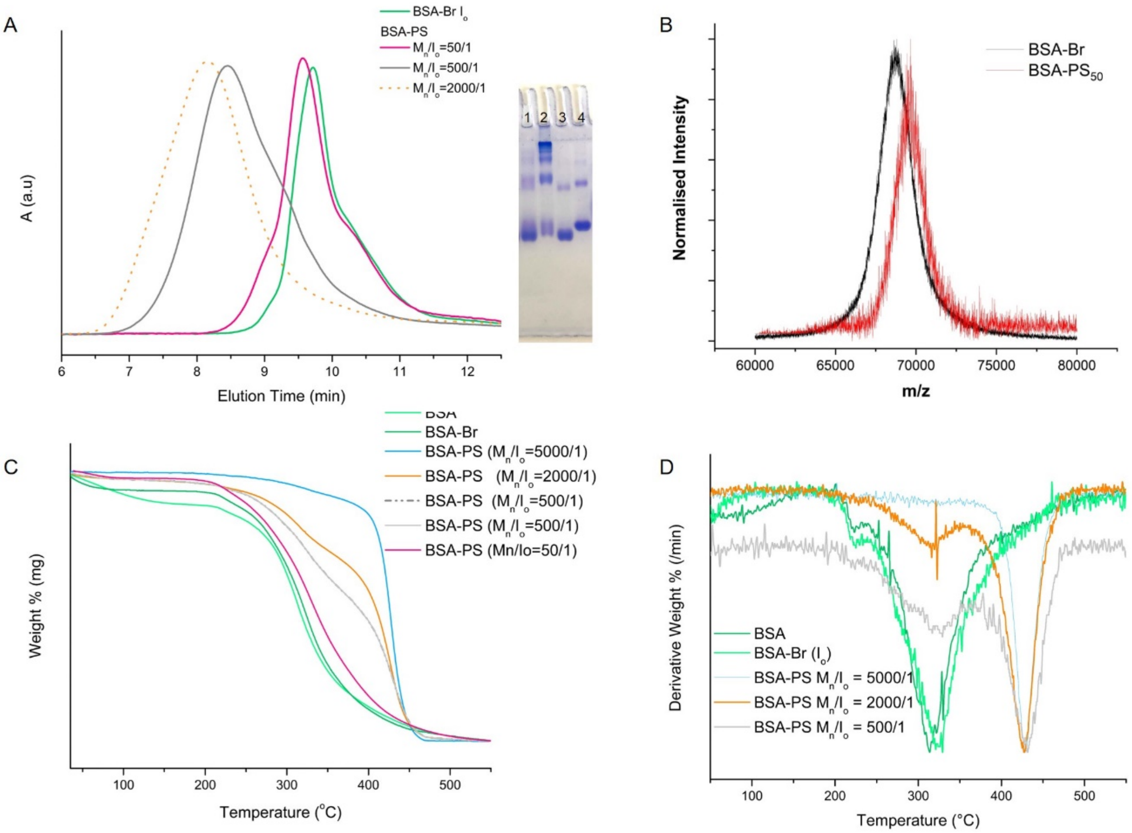

Supplementary Figure 14. Structural characterization of BSA-PS produced via oxygen tolerant photoinduced grafting from BSA-Br using feed ratio [styrene]/[BSA-Br]/[Cu^{II}]/[Me$_6$TREN] = 500/1/1.5/12 and 50/1/1.5/12 (Supplementary Table 1, Entries 10 and 11 respectively). **A)** *left:* SEC traces; *right:* PAGE lane 1: Reaction mixtures (Supplementary Table 1, Entry 11), with no products traced, lane 2: BSA-PS (Supplementary Table 1, Entry 10), lane 3: BSA-Br, lane 4: native BSA. The electrophoretic gel has been cropped for clarity; **B)** MALDI-TOF MS Spectra of BSA-Br (I$_o$) and the reaction mixture (Supplementary Table 1, Entry 11), indicating no or minimal grafting; **C)** Thermograms of BSA, BSA-Br and BSA-PS conjugates (Supplementary Table 1, Entries 4, 8, 10) and **D)** corresponding first derivatives.

Blank Experiments (Supplementary Table 1, Entries 1-3, 16, 17)
Several control experiments were performed to validate the photoinduced nature of the reaction *i.e.* the reaction did not proceed in the absence of any individual component (catalyst/ligand or initiator), in the dark at 40 °C (Supplementary Fig. 15, Supplementary Table 1, Entries 1-3) or with headspace above the reaction mixture (Supplementary Table 1, Entry 16). When the grafting of styrene from BSA-Br (I$_o$) was attempted using a 1/1 ratio between the ligand Me$_6$TREN and Cu^{II}, (Supplementary Table 1, Entry 17), no products were detected (Supplementary Fig. 15). It should be noted that special care was

taken to avoid temperature rising above 40 °C during the course of all photoinduced polymerization reactions for this reason, the blank experiments were conducted at 40 °C.

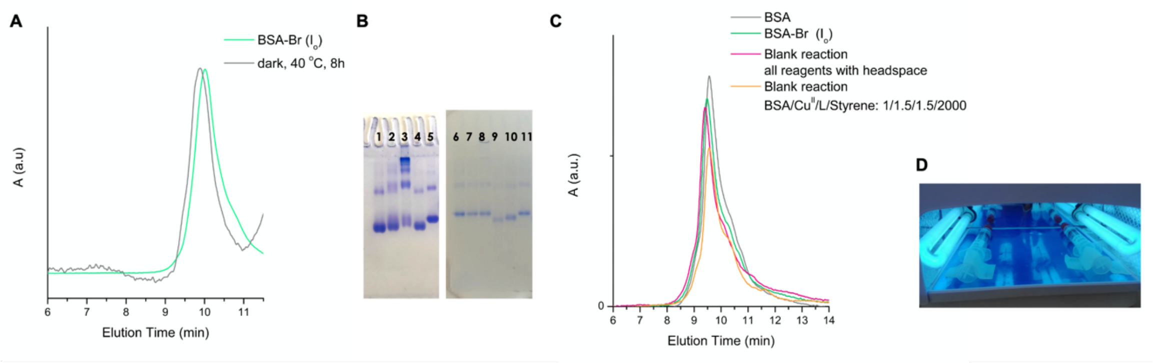

Supplementary Figure 15. Chromatographic evaluation of blank experiments. **A)** SEC traces for blank experiment in the dark (Supplementary Table 1, Entry 3); **B)** Native PAGE electrophoresis for blank experiments (Supplementary Table 1, Entries 3, 17) Native PAGE lane 1: reaction mixture from the blank experiment performed in the dark (Supplementary Table 1, Entry 3), lane 2 and 3: blank experiments not included in this study, lane 4: BSA-Br (I_o), lane 5: native BSA, lane 6: native BSA, lane 7: blank experiment using BSA/Cu^{II}/L/Styrene: 1/1.5/12/2000, lane 8: blank experiment using BSA/EBiB/Cu^{II}/L/Styrene: 1/1/1.5/12/2000, lane 9: experiment using BSA-Br and a 1/1 Cu^{II}/ Me_6TREN ratio, lane 10: BSA-Br (I_o), lane 11: native BSA. The electrophoretic gels have been cropped for clarity; **C)** SEC traces for blank experiments (Supplementary Table 1, Entries 16, 17); **D**) Experimental setup.

Supplementary Table 2. Encapsulation, substituted monomer and solvent studies

Entry	$M_n/I_o/Cu^{II}/L$[a]	Monomer M_n	Guest	Solvent	BSA-Br (I_o) Consumption
1	5000/1/1.5/12	styrene	ferritin	20 mM phosphate buffer, pH 7.4[a]	quantitative
2	2000/1/1.5/12	m-nitro styrene	n/a	20 mM phosphate buffer, pH 7.4[a]	quantitative
3	2000/1/1.5/12	p-methoxy styrene	n/a	20 mM phosphate buffer, pH 7.4[a]	quantitative
4	2000/1/1.5/12	styrene	n/a	Nanopure water	quantitative
5	2000/1/1.5/12	styrene	n/a	Tap water	quantitative
6	2000/1/1.5/12	styrene	n/a	Sea water	quantitative

M_n: monomer, I_o: initiator (BSA-Br), L: ligand (Me_6TREN).
[a] Representative reactions, all reactions were performed without using DMSO as organic cosolvent.

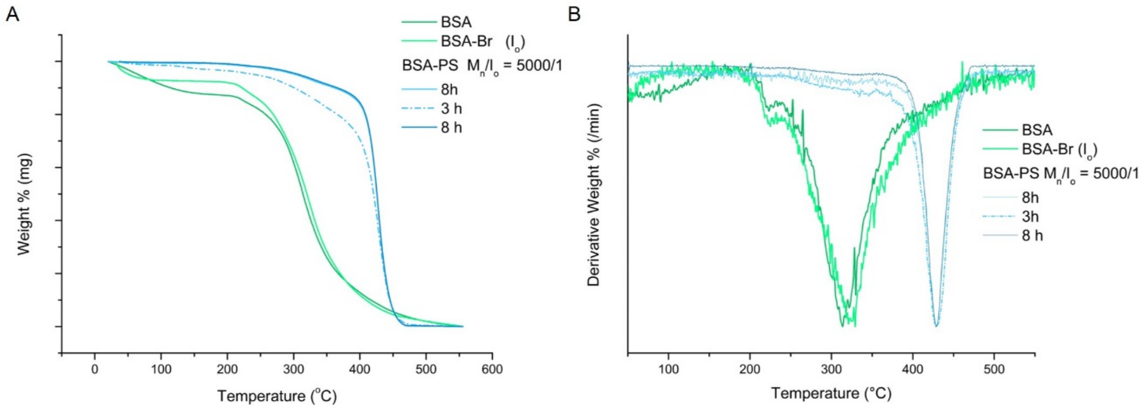

Supplementary Figure 16. Structural characterization of BSA-polymer bioconjugates. **A)** Thermograms of BSA, BSA-Br and BSA-PS conjugates (N_2 atmosphere); **B)** First derivative of the thermograms.

5. Oxygen Tolerant Photoinduced grafting of styrene from BSA-Br, in different media (Supplementary Table 2, entries 4,5,6)

The photoinduced grafting of styrene from BSA-Br was also quantitatively achieved by replacing the phosphate buffer reaction medium with either HPLC grade water (EC = 3 μS cm^{-1} at 25 °C), tap water (EC = 442 μS cm^{-1}, pH 8.0 at 25 °C) or sea water (EC = 47.2 mS cm^{-1}, pH 7.9 at 25 °C).

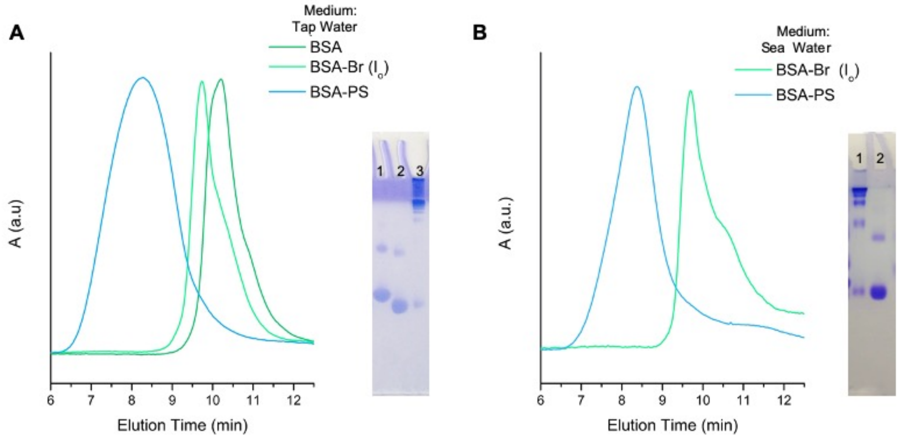

Supplementary Figure 17. Chromatographic characterization of BSA-PS bioconjugates. SEC chromatograms of BSA-PS bioconjugates produced in **A)** tap water and **B)** sea water. PAGE electrophoresis under native conditions **A)** lane 1: native BSA, lane 2: BSA-Br (I_o) in tap water, lane 3: BSA-PS in tap water (Supplementary Table 2, Entry 5); **B)** lane 1: BSA-PS in sea water (Supplementary Table 1, Entry 6), lane 2: BSA-Br (I_o) in sea water. The electrophoretic gels have been cropped for clarity.

6. Oxygen Tolerant Photoinduced RDRP grafting of substituted-styrenes from BSA-Br (Supplementary Table 2, Entries 2, 3)

All reactions were performed under the general oxygen tolerant photoinduced grafting conditions mentioned above using a feed ratio $M_n/I_o/Cu^{II}/L$ = 2000/1/1.5/12.

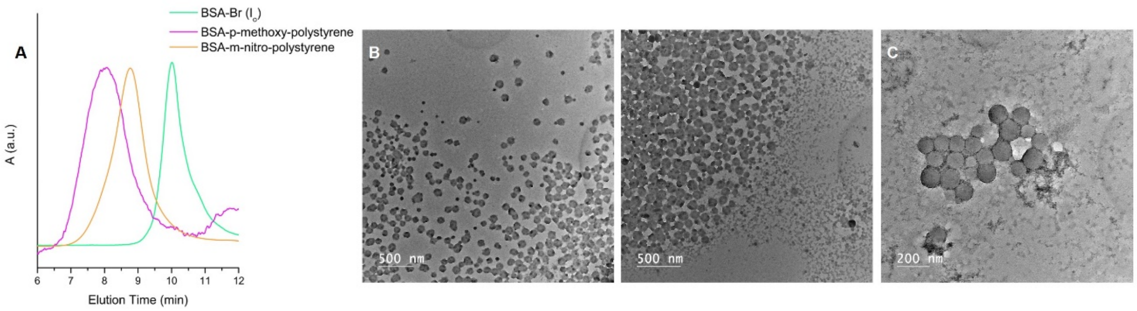

Supplementary Figure 18. Chromatographic and morphological characterization of BSA-substituted polystyrenes. **A)** SEC traces, **B)** TEM micrographs of p-methoxy substituted BSA-PS and **C)** TEM micrograph of m-nitro substituted BSA-PS.

7. BSA-pAAm Bioconjugates

All reactions were performed under the general oxygen tolerant photoinduced grafting conditions mentioned above using the feed ratios $M_n/I_o/Cu^{II}/L$ described in Supplementary Table 3. Since the monomer was soluble in the aqueous solvent, the sonication step prior

to the addition of the monomer to the biomacroinitiator was omitted. The products were characterized with native polyacrylamide electrophoresis and thermogravimetric analysis. The total volume of the reaction mixtures was kept constant via the addition of the appropriate amount of nanopure water at each variation.

Supplementary Table 3. Oxygen Tolerant Photoinduced ATRP grafting of hydrophilic monomers from BSA-Br

Entry	$M_n/I_o/Cu^{II}/L$	Monomer	Monomer (mmole)	Cu^{II} (mmole)	Cu^{II} (ppm/mM)	Me_6TREN (mmole)	Rxn time (min)
1	2000/1/0.4/2	Acrylamide	0.874	0.175×10^{-3}	6/0.009	0.874×10^{-3}	180
2	2000/1/1.5/12	Acrylamide	0.874	0.655×10^{-3}	22/0.34	5.244×10^{-3}	180
3	1500/1/1.5/12	Acrylamide	0.655	0.655×10^{-3}	22/0.34	5.244×10^{-3}	180
4	1000/1/1.5/12	Acrylamide	0.437	0.655×10^{-3}	22/0.34	5.244×10^{-3}	180
5	500/1/1.5/12	Acrylamide	0.219	0.655×10^{-3}	22/0.34	5.244×10^{-3}	180
6	2000/1/0.4/2	DMAEMA	0.874	0.175×10^{-3}	6/0.009	0.874×10^{-3}	180
7	2000/1/1.5/12	DMAEMA	0.874	0.655×10^{-3}	22/0.34	5.244×10^{-3}	180
8	1500/1/1.5/12	DMAEMA	0.655	0.655×10^{-3}	22/0.34	5.244×10^{-3}	180
9	1000/1/1.5/12	DMAEMA	0.437	0.655×10^{-3}	22/0.34	5.244×10^{-3}	180
10	500/1/1.5/12	DMAEMA	0.219	0.655×10^{-3}	22/0.34	5.244×10^{-3}	180
11	2000/1/1.5/12	$OEOA_{480}$	0.874	0.655×10^{-3}	22/0.34	5.244×10^{-3}	180
12	1000/1/1.5/12	$OEOA_{480}$	0.437	0.655×10^{-3}	22/0.34	5.244×10^{-3}	180
13	500/1/1.5/12	$OEOA_{480}$	0.219	0.655×10^{-3}	22/0.34	5.244×10^{-3}	180

M_n: monomer, I_o: initiator (BSA-Br), L: ligand (Me_6TREN), Rxn time: reaction time.
[a] Representative reactions, all reactions were performed in 20 mM phosphate buffer pH 7.4, in the absence of DMSO as organic cosolvent.

General Procedure of Oxygen Tolerant Photoinduced ATRP Grafting of Acrylamide (Am) from BSA-Br Using 6 ppm of Cu^{II} (0.09 mM), [Am]/[BSA-Br]/[Cu^{II}]/[Me_6TREN] = 2000/1/0.4/2 (Supplementary Table 3, Entry 1)

Me_6TREN (3.7 µL, 8.74 × 10^{-3} mmol, 20 equiv.) was added to 1 mL of a 0.4 mg mL^{-1} solution of $CuBr_2$ (1,75 × 10^{-3} mmol, 4 equiv.) in nanopure water to form a light blue colored solution due to the immediate copper-ligand complex formation. 100 µL of the $CuBr_2$/Me_6TREN solution (2 equiv. Me_6TREN and 0.4 $CuBr_2$ equiv., final concentration of Cu^{II}: 0.09 mM) were added to a solution consisting of acrylamide (62 mg, 0.874 mmol, 2000 equiv.) and nanopure water (0.55 mL) and immediately transferred to a 5 mL syringe equipped with a stirring bar, containing a 0.32 mM solution of the BSA-macroinitiator (**BSA-Br**) in 20 mM phosphate buffer, pH 7.4 (1.25 mL, 0.437×10^{-3} mmol). Headspace was eliminated to avoid the presence of undissolved oxygen and the reaction syringe was hermitically capped and placed under the UV light sources for 180 minutes. The reaction mixture was subsequently extensively dialyzed to eliminate the excess of the unreacted monomer and the catalyst.

General Procedure of Oxygen Tolerant Photoinduced ATRP Grafting of Acrylamide (Am) from BSA-Br [Am]/[BSA-Br]/[Cu^{II}]/[Me_6TREN] = 500-2000/1/1.5/12 (Supplementary Table 3, Entries 2-5)

Me_6TREN (14 µL, 52.44 × 10^{-3} mmol, 120 equiv.) was added to 1 mL of a 1.5 mg mL^{-1} solution of $CuBr_2$ (6.55 mmol, 15 equiv.) in nanopure water to form a light blue colored solution due to the immediate copper-ligand complex formation. 100 µL of the

$CuBr_2$/Me_6TREN solution (12 equiv. Me_6TREN and 1.5 $CuBr_2$ equiv., final concentration of Cu^{II}: 0.34 mM) were added to a solution consisting of acrylamide (500-2000 equiv.) and nanopure water (0.55 mL) and immediately transferred to a 5 mL syringe equipped with a stirring bar, containing a 0.32 mM solution of the BSA-macroinitiator (**BSA-Br**) in 20 mM phosphate buffer, pH 7.4 (1.25 mL, 0.437×10^{-3} mmol). Headspace was eliminated to avoid the presence of undissolved oxygen and the reaction syringe was hermitically capped and placed under the UV light sources for 180 minutes. The reaction mixture was subsequently extensively dialyzed to eliminate the excess of the unreacted monomer and the catalyst.

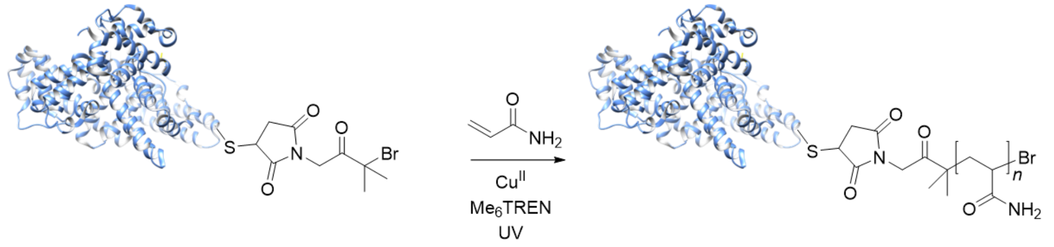

Supplementary Figure 19. Reaction scheme of the oxygen tolerant, photoinduced grafting of acrylamide from BSA-Br.

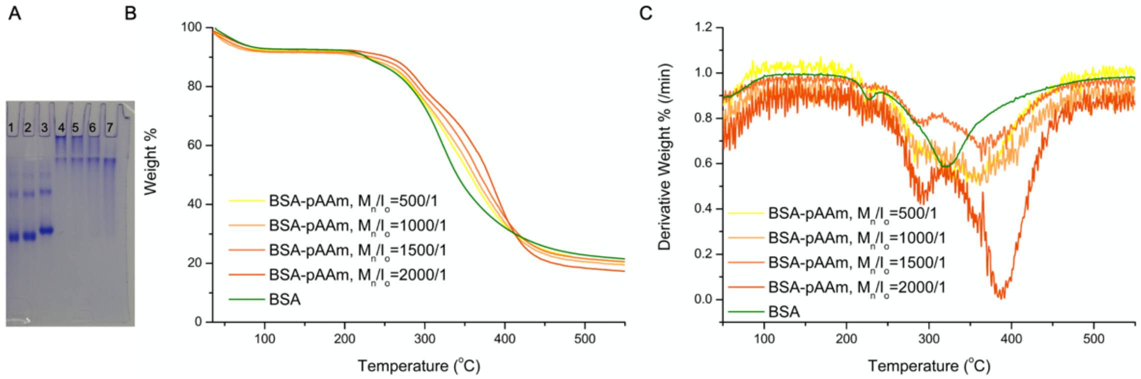

Supplementary Figure 20. Oxygen tolerant photoinduced grafting of acrylamide from BSA-Br at different feed ratios (Supplementary Table 3, Entries 1 to 5). **A)** PAGE lane 1: reaction with 2000 eq. Am, using 6 ppm Cu (Supplementary Table 3, Entry 1), with no products traced, lane 2: BSA-Br (I_o), lane 3: native BSA, lane 4: reaction with 2000 eq. Am (Supplementary Table 3, Entry 2), lane 5: reaction with 1500 eq. Am (Supplementary Table 3, Entry 3), lane 6: reaction with 1000 eq. Am (Supplementary Table 3, Entry 4), lane 7: reaction with 500 eq. Am (Supplementary Table 3, Entry 5). The electrophoretic gel has been cropped for clarity; **B)** Thermograms of BSA and BSA-pAAm conjugates (Supplementary Table 3, Entries 1-5) and **C)** corresponding first derivatives.

8. BSA-POEOA Bioconjugates

All reactions were performed under the general oxygen tolerant photoinduced grafting conditions mentioned above using the feed ratios $M_n/I_o/Cu^{II}/L$ described in Supplementary Table 3 (Entries 11-13). Since the monomer was soluble in the aqueous solvent, the sonication step prior to the addition of the monomer to the biomacroinitiator was omitted. The products were characterized with native polyacrylamide electrophoresis and aqueous SEC. The total volume of the reaction mixtures was kept constant via the addition of the appropriate amount of nanopure water at each variation.

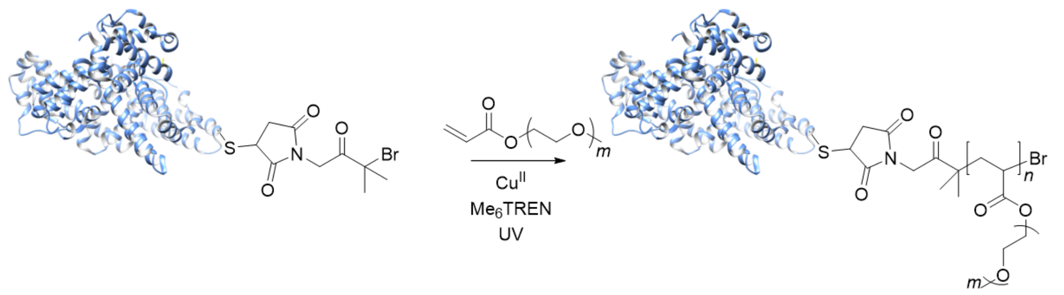

Supplementary Figure 21. Reaction scheme of the oxygen tolerant, photoinduced grafting of $OEOA_{480}$ from BSA-Br.

General Procedure of Oxygen Tolerant Photoinduced ATRP Grafting of Oligo (ethylene oxide) Methyl Ether Acrylate ($OEOA_{480}$) from BSA-Br, [OEOA]/[BSA-Br]/[Cu^{II}]/[Me_6TREN] = 500-2000/1/1.5/12 (Supplementary Table 3, Entry 11-13)

Me_6TREN (14 µL, 52.44 × 10^{-3} mmol, 120 equiv.) was added to 1 mL of a 1.5 mg mL^{-1} solution of $CuBr_2$ (6.55 mmol, 15 equiv.) in nanopure water to form a light blue colored solution due to the immediate copper-ligand complex formation. 100 µL of the $CuBr_2$/Me_6TREN solution (12 equiv. Me_6TREN and 1.5 $CuBr_2$ equiv., (final concentration of Cu^{II}: 0.34 mM) were added to a solution consisting of $OEOA_{480}$ (385 µL, 0.874 mmol, 2000 equiv.) and nanopure water (165 µL) and immediately transferred to a 5 mL syringe equipped with a stirring bar, containing a 0.32 mM solution of the BSA-macroinitiator (**BSA-Br**) in 20 mM phosphate buffer, pH 7.4 (1.25 mL, 0.437×10^{-3} mmol). Headspace was eliminated to avoid the presence of undissolved oxygen and the reaction syringe was hermitically capped and placed under the UV light sources for 180 minutes. The reaction mixture was subsequently extensively dialyzed to eliminate the excess of the unreacted monomer and the catalyst.

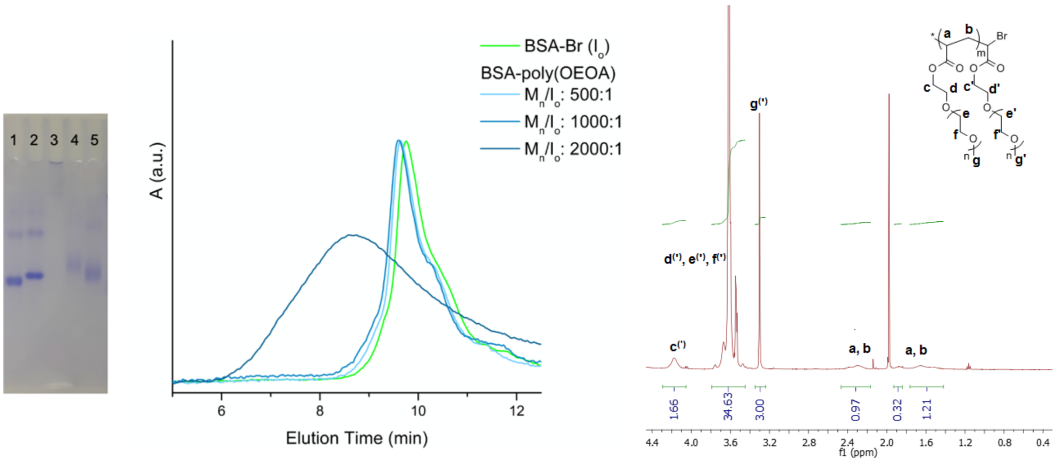

Supplementary Figure 22. Characterization of BSA-poly(OEOA). *Left:* PAGE lane 1: native BSA, lane 2: BSA-Br (I_o), lane 3: reaction with 2000 eq. OEOA (Supplementary Table 3, Entry 11), lane 4: reaction with 1000 eq. OEOA (Supplementary Table 3, Entry 12), lane 5: reaction with 500 eq. OEOA (Supplementary Table 3, Entry 13). The electrophoretic gel has been cropped for clarity; *Middle:* SEC chromatography, Green trace: BSA-Br (I_o), Dark blue trace: BSA-poly(OEOA); *Right*: ^{1}H-NMR spectrum of BSA-poly(OEOA) at D_2O.

9. BSA-PDMAEMA Bioconjugates

All reactions were performed under the general oxygen tolerant photoinduced grafting conditions mentioned above using the feed ratios $M_n/I_o/Cu^{II}/L$ described in Supplementary Table 3 (Entries 6-10). Since the monomer was soluble in the aqueous solvent, the sonication step prior to the addition of the monomer to the biomacroinitiator was omitted. The products were characterized with native polyacrylamide electrophoresis and thermogravimetric analysis. The total volume of the reaction mixtures was kept constant via the addition of the appropriate amount of nanopure water at each variation.

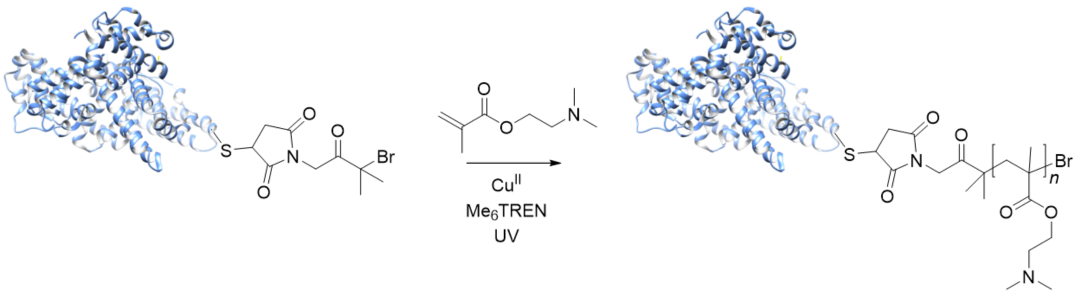

Supplementary Figure 23. Reaction scheme of the oxygen tolerant, photoinduced grafting of acrylamide from BSA-Br.

General Procedure of Oxygen Tolerant Photoinduced ATRP grafting of 2-(Dimethylamino) Ethyl Methacrylate (DMAEMA) from BSA-Br Using 6 ppm of Cu^{II}

(0.09 mM), [DMAEMA]/[BSA-Br]/[Cu^{II}]/[Me$_6$TREN] = 2000/1/0.4/2 (Supplementary Table 3, Entry 6)

Me$_6$TREN (3.7 µL, 8.74 × 10^{-3} mmol, 20 equiv.) was added to 1 mL of a 0.4 mg mL^{-1} solution of CuBr$_2$ (1,75 × 10^{-3} mmol, 4 equiv.) in nanopure water to form a light blue colored solution due to the immediate copper-ligand complex formation. 100 µL of the CuBr$_2$/Me$_6$TREN solution (2 equiv. Me$_6$TREN and 0.4 CuBr$_2$ equiv., final concentration of Cu^{II}: 0.09 mM) were added to a solution consisting of DMAEMA (147 µL, 0.874 mmol, 2000 equiv.) and nanopure water (403 µL) and immediately transferred to a 5 mL syringe equipped with a stirring bar, containing a 0.32 mM solution of the BSA-macroinitiator (**BSA-Br**) in 20 mM phosphate buffer, pH 7.4 (1.25 mL, 0.437×10^{-3} mmol). Headspace was eliminated to avoid the presence of undissolved oxygen and the reaction syringe was hermitically capped and placed under the UV light sources for 180 minutes. The reaction mixture was subsequently extensively dialyzed to eliminate the excess of the unreacted monomer and the catalyst.

General Procedure of Oxygen Tolerant Photoinduced ATRP grafting of 2-(Dimethylamino) Ethyl Methacrylate (DMAEMA) from BSA-Br [DMAEMA]/[BSA-Br]/[Cu^{II}]/[Me$_6$TREN] = 2000/1/1.5/12 (Supplementary Table 3, Entry 7-10)

Me$_6$TREN (14 µL, 52.44 × 10^{-3} mmol, 120 equiv.) was added to 1 mL of a 1.5 mg mL^{-1} solution of CuBr$_2$ (6.55 mmol, 15 equiv.) in nanopure water to form a light blue colored solution due to the immediate copper-ligand complex formation. 100 µL of the CuBr$_2$/Me$_6$TREN solution (12 equiv. Me$_6$TREN and 1.5 CuBr$_2$ equiv., final concentration of Cu^{II}: 0.34 mM) were added to a solution consisting of DMAEMA (147 µL, 0.874 mmol, 2000 equiv.) and nanopure water (403 µL) and immediately transferred to a 5 mL syringe equipped with a stirring bar, containing a 0.32 mM solution of the BSA-macroinitiator (**BSA-Br**) in 20 mM phosphate buffer, pH 7.4 (1.25 mL, 0.437×10^{-3} mmol). Headspace was eliminated to avoid the presence of undissolved oxygen and the reaction syringe was hermitically capped and placed under the UV light sources for 180 minutes. The reaction mixture was subsequently extensively dialyzed to eliminate the excess of the unreacted monomer and the catalyst.

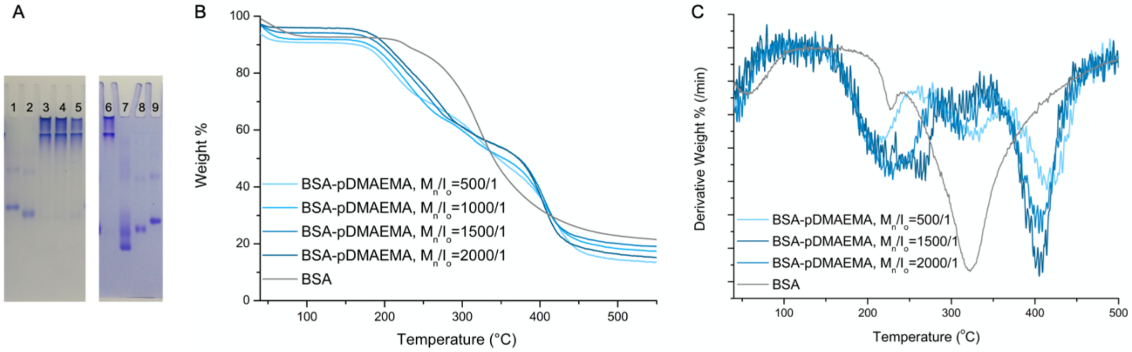

Supplementary Figure 24. Structural characterization of BSA-PDMAEMA produced via oxygen tolerant photoinduced grafting of DMAEMA from BSA-Br at different feed ratios (Supplementary Table 3, Entries 6 to 10). **A)** Native PAGE lane 1: native BSA, lane 2: BSA-Br (I$_o$), lane 3: reaction with 2000 eq. DMAEMA (Supplementary Table 3, Entry 7), lane 4: reaction with 1500 eq. DMAEMA (Supplementary Table 3, Entry 8), lane 5: reaction with 1000 eq. DMAEMA (Supplementary Table 3, Entry 9), lane 6: reaction with 2000 eq. DMAEMA using 6 ppm Cu^{II} (Supplementary Table 3, Entry 6), lane 8: native BSA, lane 9: BSA-Br (I$_o$); The electrophoretic gels have been cropped for clarity; **B)**

Thermograms of BSA and BSA-PDMAEMA conjugates (Supplementary Table 3, Entries 7-10) and **C)** corresponding first derivatives.

PDMAEMA, is a multi-responsive polymer that responds both to pH and temperature under defined conditions. This behavior was observed during our studies (Supplementary Fig. 20). More specifically, PDMAEMA conveyed its temperature and pH response[7] to the protein-polymer bioconjugate (Supplementary Fig. 25). The assembled BSA-PDMAEMA nanostructures were imaged with TEM (TEM samples were stained with uranyl acetate prior observation).

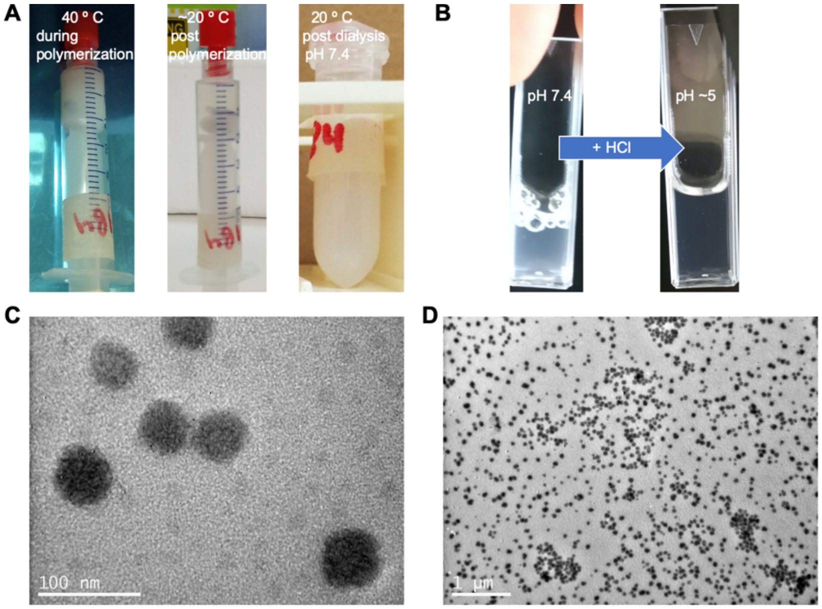

Supplementary Figure 25. Responsive behavior and morphological characterization of BSA-PDMAEMA. **A)** Photographs of the opaque BSA-PDMAEMA solution at slightly elevated temperature (~ 40 °C), transparent BSA-PDMAEMA solution at room temperature (~ 40 °C). pH change via dialysis resulted in an opaque solution; **B)** Acidification with HCl (pH 5.0) resulted in an opaque BSA-PDMAEMA solution; **C)** and **D)** TEM micrographs of BSA-PDMAEMA nanoparticles.

10. BSA-PS nanocarriers

Encapsulation experiments (Supplementary Table 2, Entry 1)

A solution consisting of styrene (0.25 mL, 2.185 mmol, 5000 equiv.) and nanopure water (0.3 mL) was sonicated for 30 sec to form an emulsion. Me_6TREN (14 µL, 52.44 × 10^{-3} mmol, 120 equiv.) was added to 1 mL of a 1.5 mg mL^{-1} solution of $CuBr_2$ (6.55 mmol, 15 equiv.) in nanopure water to form a light blue colored solution due to the immediate copper-ligand complex formation. 100 µl of the $CuBr_2/Me_6TREN$ solution (12 equiv. Me_6TREN and 1.5 $CuBr_2$ equiv.) were added to the monomer emulsion and immediately transferred to a 5 mL syringe equipped with a stirring bar, containing a 0.32 mM solution of the BSA-macroinitiator (BSA-Br) in 20 mM phosphate buffer, pH 7.4 (1.25 mL, 0.437×10^{-3} mmol) and 60 µl of 50-150 mg ml^{-1} ferritin from equine spleen Type I, 150 mM saline solution (Sigma). Headspace was eliminated to avoid the presence of undissolved

oxygen and the reaction syringe was hermitically capped and placed under the UV or other light sources for specified amounts of time. Dialysis or removal of the monomer under reduced pressure preceded chromatography in all aliquots withdrawn from the reaction vessel for SEC and PAGE analysis. A further purification step was added to remove non-encapsulated ferritin briefly, the reaction mixtures were centrifuged (10,000 rpm), the supernatant removed and the precipitated nanoparticles resuspended in 20 mM phosphate buffer thrice before analysing with SEC or imaging with microscopy.

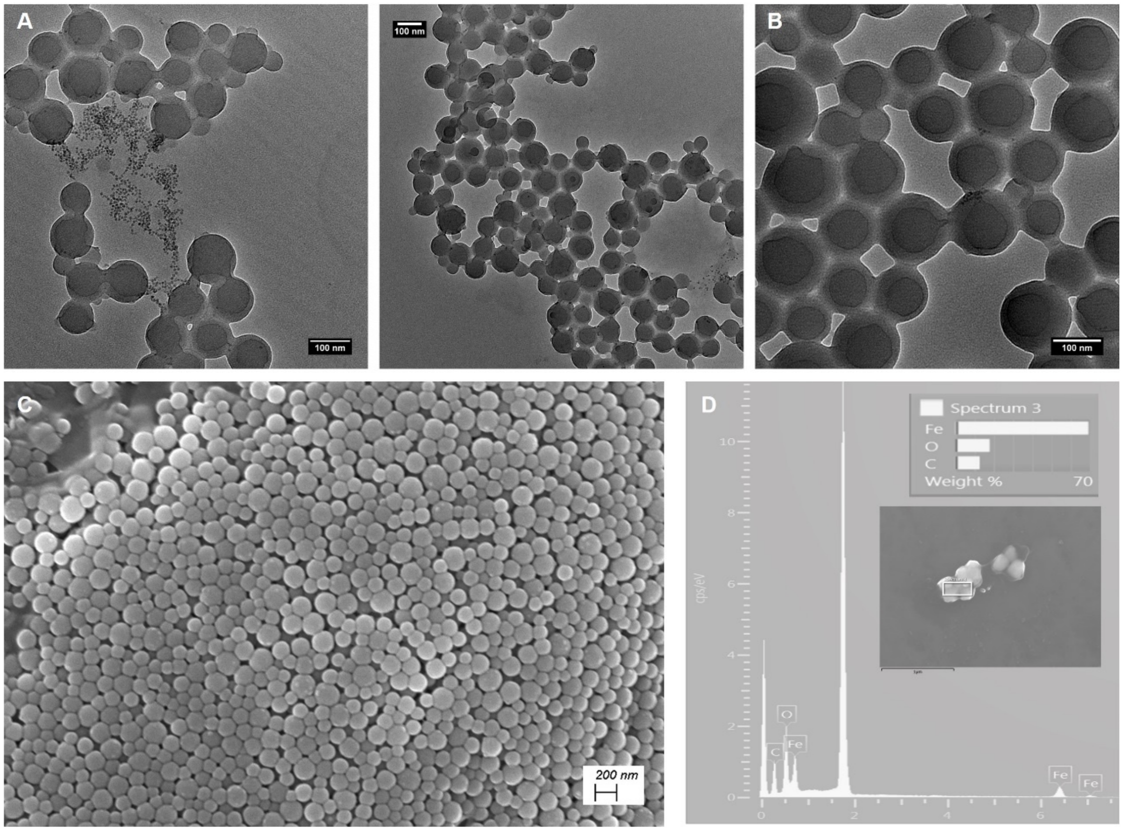

Supplementary Figure 26. Morphological characterization of BSA-PS, ferritin nanocarriers. TEM micrographs of BSA-PS, ferritin nanocarriers **A)** prior and **B)** post purification; **C)** SEM micrograph and **D)** EDX analysis of BSA-PS, ferritin nanocarriers.

11. BSA-PS esterase-like activity[8]

32 mg of p-nitrophenyl acetate (pNPA) were dissolved in 600 µL MeOH to form a 300 mM stock solution which was fractionated and stored at -20 °C. 21 µL of a 0.35 mM BSA solution were diluted with 20 mM phosphate buffer 7.4 to form a 3 µM protein solution (2.4 mL). Immediately prior to measurement, 6 µL of the 300 mM pNPA solution in methanol were diluted with nanopure water to form 0.6 mL of a 3 mM pNPA solution. The reaction was initiated by the addition of the 3 mM pNPA solution to the BSA solution and the esterase-like activity of BSA was monitored by UV at 400 nm. The ability of BSA-Br (I_o) and BSA-PS to hydrolyze pNPA was also tested following the same protocol. A blank experiment was performed using the same protocol in the absence of the protein.

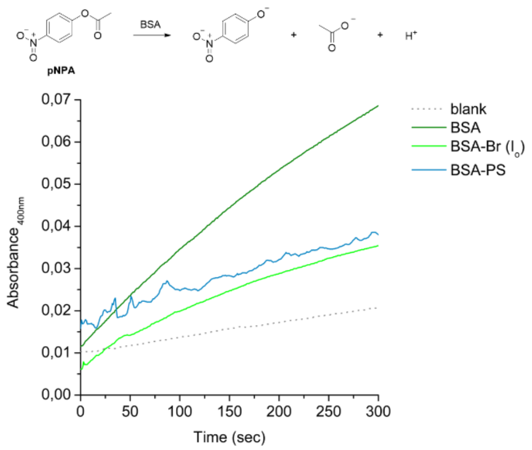

Supplementary Figure 27. Esterase-like activity of BSA and BSA-bioconjugates.

Supplementary Table 4. Oxygen Tolerant Photoinduced ATRP Grafting from HAS, GOx and β-gal

Entry	$M_n/I_o/Cu^{II}/L$	Protein	Monomer	Monomer (mmole)	Cu^{II} (mmole)	Cu^{II} (ppm/mM)	Me_6TREN (mmole)
1	2000/1/1.5/12	HSA	Styrene	0.874	0.655×10^{-3}	22/0.34	5.244×10^{-3}
2	2000/1/1.5/12	GOx	Styrene	0.874	0.655×10^{-3}	22/0.34	5.244×10^{-3}
3	2000/1/1.5/12	β-gal	Am	0.874	0.655×10^{-3}	22/0.34	5.244×10^{-3}
4	2000/1/1.5/12	β-gal	DMAEMA	0.874	0.655×10^{-3}	22/0.34	5.244×10^{-3}

M_n: monomer, I_o: initiator (BSA-Br), L: ligand (Me_6TREN)
[a] Representative reactions, all reactions were performed in 20 mM phosphate buffer pH 7.4, in the absence of DMSO as organic cosolvent.

12. Human Serum Albumin (HSA) Bioconjugates

Synthesis of the HSA-macroinitiator (I_o)

A 150 mM solution of 2-bromo-2-methyl-propionic acid 2-(2,5-dioxo-2,5-dihydro-pyrrol-1-yl)-ethyl ester **4** in DMSO (20 eq.) was slowly added to 9.0 mL of a 0.35 mM solution of native HSA (1 eq.) in 20 mM phosphate buffer (pH 7.4). The reaction mixture was gently shaken for 48 hours at 7°C. To eliminate the excess of **4**, the mixture was subsequently extensively dialyzed initially against 10% DMSO in 5 mM phosphate buffer pH 7.4 and then twice against 20 mM phosphate buffer pH 7.4 using regenerated cellulose dialysis membranes with a MWCO of 10 kDa. The macroinitiator was characterized by SDS gel electrophoresis (see Supplementary Fig. 29, **A**) and SEC chromatography (Eluent: phosphate buffer 5 mM pH 7.4, 10 % acetonitrile, room temperature, Column: SEC-300 BioBasic, flow rate: 1.0 mL·min^{-1}, see Supplementary Fig. 29, **B**). SEC samples were prepared by dissolving 50 µL of the HSA-macroinitiator solution in 950 µL 10% MeCN in 5% phosphate buffer, pH 7.4. The HSA-macroinitiator **(HSA-Br, I_o)** solution was stored at 4 ºC.

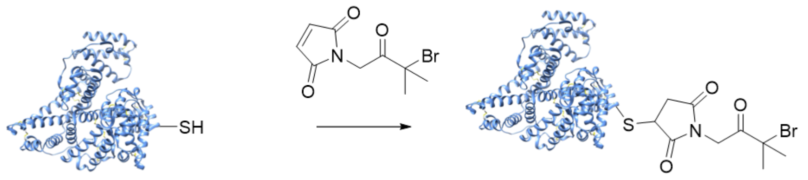

Supplementary Figure 28. Synthesis of the biomacroinitiator HSA-Br (I_o).

Synthesis of HSA-polymer bioconjugates via Oxygen Tolerant Photoinduced RDRP

The reaction was performed under the general oxygen tolerant photoinduced grafting conditions, using feed as mentioned above using the feed ratio $M_n/I_o/Cu^{II}/L$ = 2000/1/1.5/12 as described in Supplementary Table 4 (Entry 1). The products were characterized with native polyacrylamide electrophoresis and aqueous SEC and imaged with SEM.

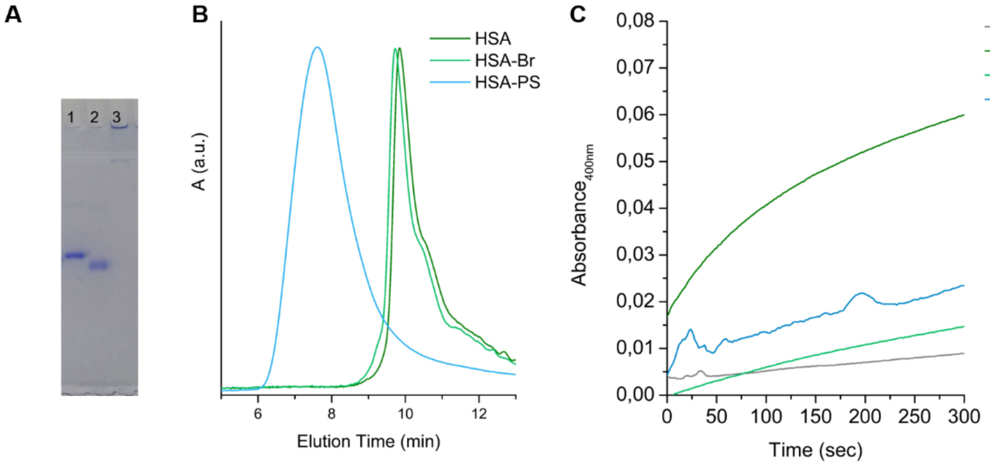

Supplementary Figure 29. Characterization of HSA-PS biohybrids. **A)** Native PAGE, lane 1: native HSA, lane 2: HSA-Br, lane 3: HSA-PS. The electrophoretic gel has been cropped for clarity; **B)** SEC chromatography of HSA, HSA-Br and HSA-PS; **C)** Esterase-like activity of HSA and HSA-bioconjugates.

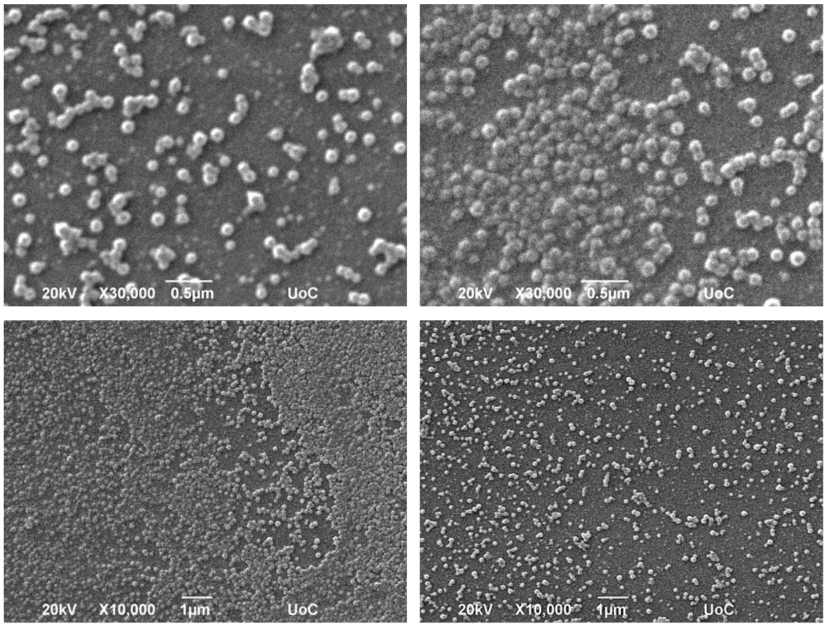

Supplementary Figure 30. Morphological characterization of HSA-PS biohybrids. SEM micrographs of HSA-PS amphiphiles (Supplementary Table 4, Entry 1).

13. beta-Galactosidase Bioconjugates

Synthesis of *N*-hydroxysuccinimide-2-bromo-2-methylpropionate (5)[9]

N-Hydroxysuccinimide (575 mg, 5.0 mmol) and triethylamine (1.4 mL, 10 mmol) were dissolved in 100 mL dichloromethane under nitrogen atmosphere, in a 250 ml round-bottomed flask equipped with a magnetic stirrer. The flask was cooled to 0°C and 2-bromo-2-methylpropionyl bromide (0.68 mL, 5.5 mmol) was added dropwise. The mixture was stirred for 45 min at 0 °C and allowed to reach room temperature. After 2 hours, the reaction mixture was poured into an excess of cold water and extracted with diethyl ether (3 x 15 mL). The organic layer was washed with a saturated aqueous solution of sodium carbonate (3 x 15 mL), diluted HCl aqueous solution (pH 4.5, 3 x 10 mL), and again with saturated aqueous solution of sodium carbonate (3 x 15 mL). The organic layer was dried over anhydrous magnesium sulphate, filtered and the solvent removed under reduced pressure to afford the product **5** as a white solid (498 g, 1.89 mmol, 37.7%).

^{1}H NMR ($CDCl_3$) δ (ppm) 2.08 (s, 6H, C(C**H**$_3$)$_2$Br), 2.87 (s, 4H, succ).
^{13}C {^{1}H} NMR ($CDCl_3$) δ (ppm) 25.61 (2C, C_{succ}), 30.70 (2C, C(**C**H$_3$)$_2$Br), 51.12 (1C, **C**(CH$_3$)$_2$Br), 167.48 (1C, **C**=O), 168.52 (2C, **C**$_{succ}$=O).

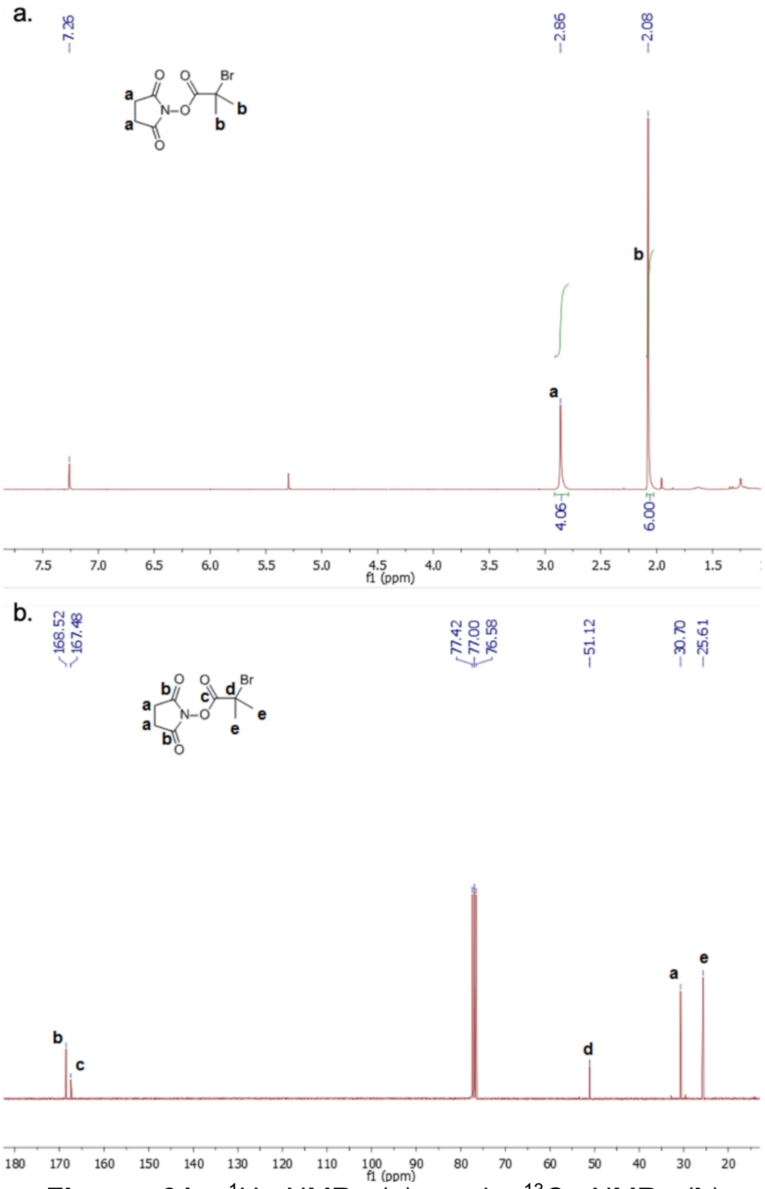

Supplementary Figure 31. ^{1}H NMR (**a**) and, ^{13}C NMR (**b**) spectra of *N*-hydroxysuccinimide-2-bromo-2-methylpropionate (**5**).

Synthesis of the β-galactosidase-macroinitiator (I_o)

Following a modified literature protocol[10] a 3.5 mM solution of *N*-hydroxysuccinimide-2-bromo-2-methylpropionate **5** in DMSO (0.5 mL, 1 eq) was slowly added to 5.0 mL of a 0.35 mM solution of native β-gal (1 eq.) in 20 mM phosphate buffer (pH 7.4), 1 mM $MgCl_2$. The reaction mixture was gently shaken for 6 hours at 7°C. The macroinitiator was characterized by native gel electrophoresis (see Supplementary Fig. 33 *left*). The β-galactosidase-macroinitiator **(β-gal-Br, I_o)** solution was stored at 4 ºC.

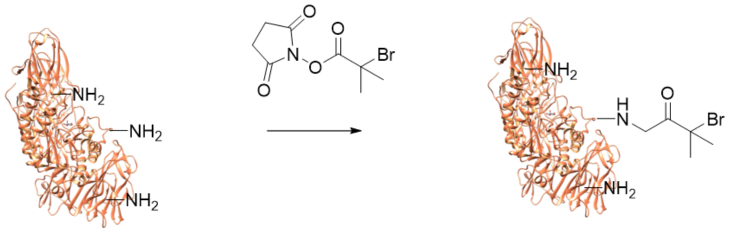

Supplementary Figure 32. Synthesis of the *β*-galactosidase-macroinitiator.

Synthesis of beta-Galactosidase-polymer bioconjugates via Oxygen Tolerant Photoinduced RDRP

The reaction was performed under the general oxygen tolerant photoinduced grafting conditions, using feed as mentioned above using the feed ratio $M_n/I_o/Cu^{II}/L$ = 2000/1/1.5/12 as described in Supplementary Table 4 (Entries 2 and 3). The products were characterized with native polyacrylamide electrophoresis (Supplementary Fig. 33).

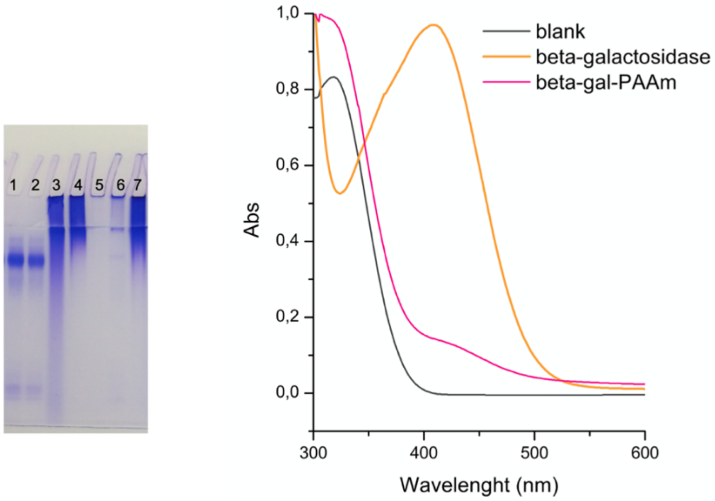

Supplementary Figure 33. Characterization of beta-galactosidase bioconjugates. *Left:* Native PAGE lane 1: native *β*-gal, lane 2: *β*-gal-Br (I_o), lane 3: *β*-gal-PDMAEMA (not included in this study); lane 4: *β*-gal-PDMAEMA (Supplementary Table 4, Entry 3); lane 5: *β*-gal-PAAm (Supplementary Table 4, Entry 4), lane 6: *β*-gal-PAAm (Supplementary Table 4, Entry 5), lane 7: *β*-gal-PDMAEMA (Supplementary Table 4, Entry 2). The electrophoretic gel has been cropped for clarity; *Right:* Enzymatic kinetic measurement of *β*-gal, *β*-gal-PAAm; catalytic activity measured at 420 nm.

Catalytic activity of β-galactosidase-PAAm[11]

400 µL of a 0.025 mM solution of *β*-galactosidase were added in 2.2 mL 100 mM phosphate buffer pH 7.4, 1 mM $MgCl_2$. The reaction was initiated by the addition of 400 µL of a 2.5 mM stock solution of the substrate *o*-Nitrophenyl-*β*-D-galactopyranoside (ONPG) and monitored by UV at 420 nm. (Supplementary Fig. 33, *right*)

To evaluate the catalytic activity of *β*-gal-Br (I_o) and *β*-gal-PAAm the same protocol was followed. A blank experiment was performed using the same protocol in the absence of the protein.

14. Glucose Oxidase Bioconjugates

Synthesis of the GOx-macroinitiator (I_o)
Following a modified literature protocol[10] 3.5 mM solution of *N*-hydroxysuccinimide-2-bromo-2-methylpropionate **5** in DMSO (0.5 mL, 1 eq) was slowly added to 5.0 mL of a 0.35 mM solution of native GOx (1 eq.) in 20 mM phosphate buffer (pH 7.4). The reaction mixture was gently shaken for 6 hours at 7°C. The mixture was subsequently extensively dialyzed initially against 10% DMSO in 5 mM phosphate buffer pH 7.4 and then twice against 20 mM phosphate buffer pH 7.4 using regenerated cellulose dialysis membranes with a MWCO of 10 kDa. The macroinitiator was characterized by native gel electrophoresis (see Supplementary Fig. 35 *left*) and SEC chromatography (Eluent: phosphate buffer 5 mM pH 7.4, 10% acetonitrile, room temperature, Column: SEC-300 BioBasic, flow rate: 1.0 mL·min^{-1}, see Supplementary Fig. 35 *right*). SEC samples were prepared by dissolving 50 µL of the BSA-macroinitiator solution in 950 µL 10% MeCN in 5% phosphate buffer, pH 7.4. The GOx-macroinitiator **(GOx-Br, I_o)** solution was stored at 4 ºC.

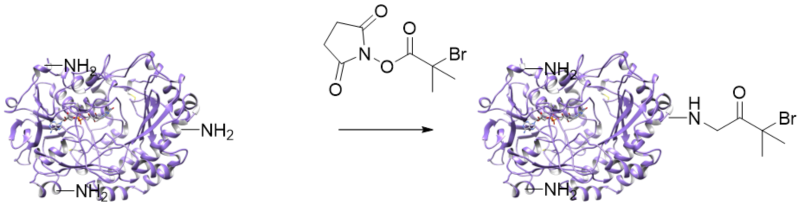

Supplementary Figure 34. Synthesis of the GOx-macroinitiator.

Synthesis of GOx-polymer bioconjugates via Oxygen Tolerant Photoinduced RDRP
The reaction was performed under the general oxygen tolerant photoinduced grafting conditions, using feed ratio styrene/GOx-Br/Cu^{II}/L = 2000/1/1.5/12 as described in Supplementary Table 4 (Entry 2). The products were characterized with native polyacrylamide electrophoresis and aqueous SEC and imaged with SEM and TEM.

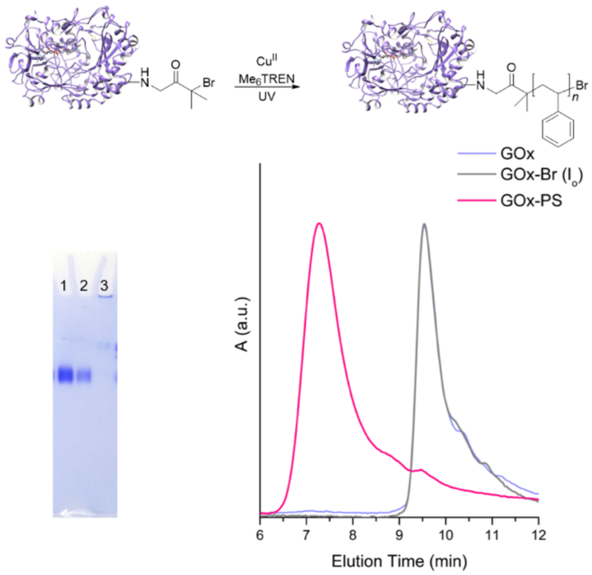

Supplementary Figure 35. Grafting of styrene from GOx-macroinitiator and chromatographic characterization of GOx-PS biohybrids. *Top:* Schematic representation of the polymerization; *Bottom Left:* Native PAGE lane 1: native GOx, lane 2: GOx-Br (I_o), lane 3: reaction with 2000 eq. styrene (Supplementary Table 4, Entry 1). The electrophoretic gel has been cropped for clarity; *Bottom Right:* SEC chromatography, Grey trace: GOx, Purple trace: GOx-Br (I_o), Pink trace: GOx-PS.

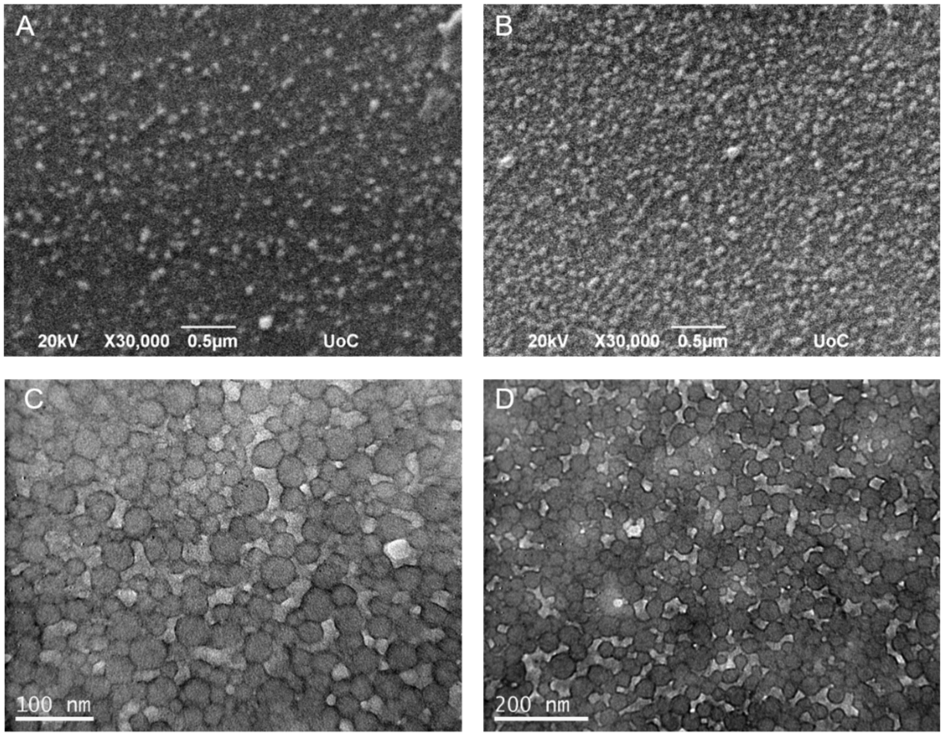

Supplementary Figure 36. Morphological characterization of GOx-PS. **A, B)** SEM and **C, D)** TEM micrographs of GOx-PS synthesized using molar feed ratio $[Mn]/[I_o]$=2000/1 (Supplementary Table 4, Entry 2).

Catalytic activity of GOx-PS[12]

A recently developed methodology entailing enzyme-catalysis-induced production of Prussian blue nanoparticles was employed as a simple colorimetric GOx assay. Briefly, 150 µL of a 22 mM glucose solution was added at the mixture of 1.5 mL of 0.5 mM $K_3Fe(CN)_6$ and 1.5 mL of 0.5 mM $FeCl_3$ solution in 50 mM Na_2HPO_4-citric acid, pH 3.0 in plastic cuvette. The reaction was initiated by the addition of 150 µL of a 0.015 mM Gox solution and an enzymatic kinetic measurement was carried out by monitoring the absorbance change at 706 nm. The catalytic profiles of GOx-Br (I_o) and GOx-PS were also monitored following the same protocol. A blank experiment was performed using the same protocol in the absence of the protein.

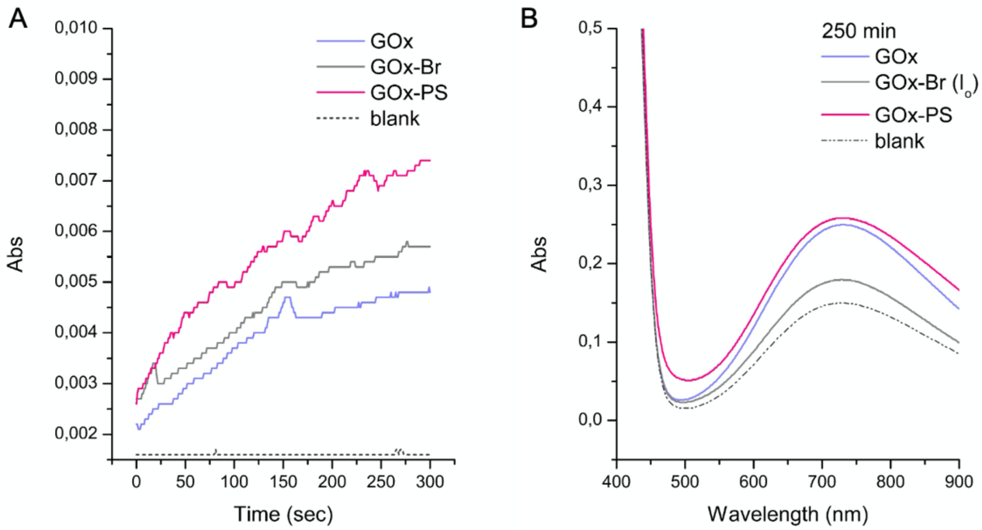

Supplementary Figure 37. Catalytic activity of GOx-PS biohybrids measured at 706 nm. **A)** Enzymatic kinetic initial activity measurement of GOx, GOx-Br (I_o) and GOx-PS catalytic activity; **B)** spectra of enzymatic reaction mixtures after 250 minutes.

15. SDS-PAGE Electrophoresis

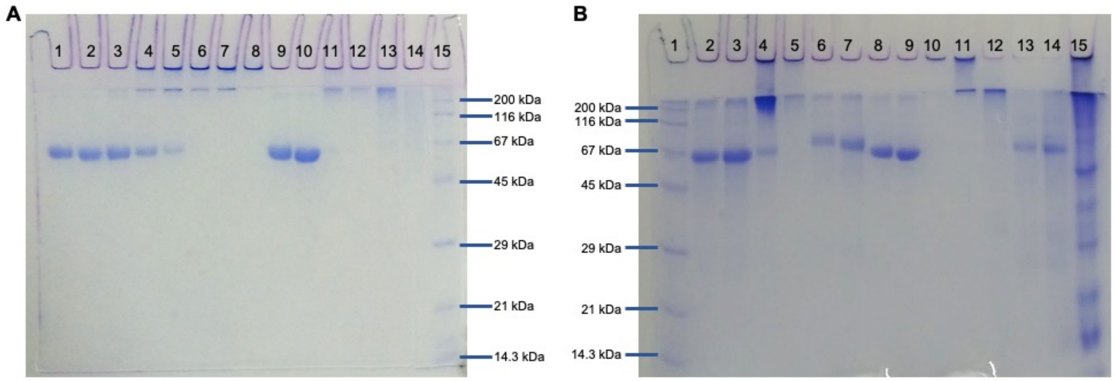

Supplementary Figure 38. SDS-PAGE electrophoresis of BSA and BSA bioconjugates. **(A)** Time course of [styrene]/[BSA-Br]/[Cu^{II}]/[Me_6TREN] = 2000/1/1.5/12 reaction (Supplementary Table 1, Entry 9), lane 1: 5min, lane 2: 15 min, lane 3: 35 min, lane 4: 45 min, lane 5: 70 min, lane 6: 100 min, lane 7: 130 min, lane 8: 300 min, lane 9: BSA-Br (I_o), lane 10: native BSA, lane 11: BSA-PAAm [Am]/[BSA-Br]=2000/1, lane 12: BSA-PAAm [Am]/[BSA-Br]=1500/1, lane 13: BSA-PAAm [Am]/[BSA-Br]=1000/1, 14: BSA-PAAm [Am]/[BSA-Br]=500/1, lane 14: Molecular Weight Markers; **(B)** lane 1: Molecular Weight Markers, lanes 2 and 3: blank reactions (with headspace), lane 4: BSA-DMAEMA 6 ppm Cu^{II}, lane 5: BSA-POEOA [OEOA]/[BSA-Br]=2000/1, lane 6: BSA-POEOA [OEOA]/[BSA-Br]=1000/1, lane 7: BSA-POEOA [OEOA]/[BSA-Br]=500/1, lane 8: BSA-Br (I_o), lane 9: native BSA, lane 10: BSA-PS [Styrene]/[BSA-Br]=2000/1, lane 11: BSA-PS [Styrene]/[BSA-Br]=1000/1, lane 12: BSA-PS [Styrene]/[BSA-Br]=500/1, lane 13: BSA-PS [Styrene]/[BSA-Br]=50/1, lane 14: BSA-PS [Styrene]/[BSA-Br]=50/1, lane 15: Molecular Weight Markers.

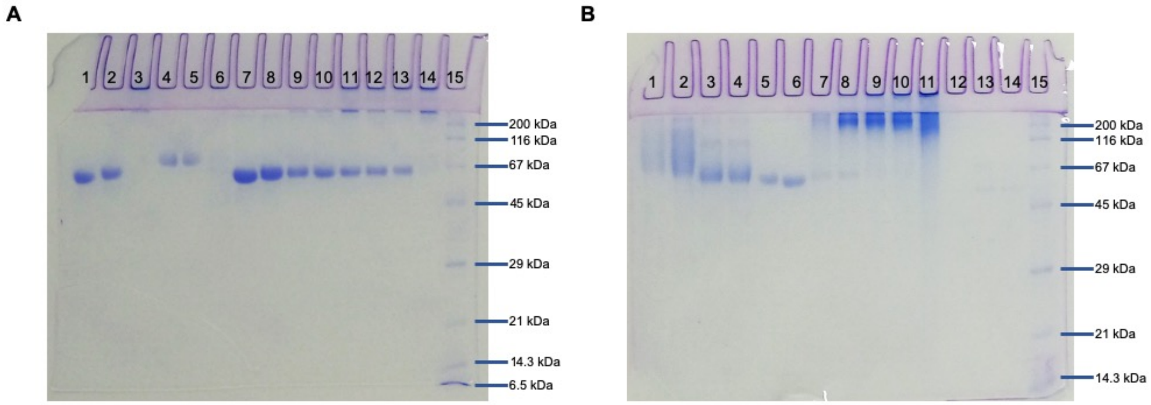

Supplementary Figure 39. SDS-PAGE electrophoresis of protein-polymer bioconjugates. **(A)** lane 1: native HSA, lane 2: HSA-Br, lane 3: HSA-PS, lane 4: native GOx, lane 5: GOx -Br, lane 6: GOx -PS lane 7: native BSA, lane 8: BSA-Br, lanes 9-14: ON/OFF time course of [styrene]/[BSA-Br]/[Cu^{II}]/[Me_6TREN] = 2000/1/1.5/12 reaction (Table 1, Entry 5), lane 9: 35 min ON, lane 10: 2 hours OFF, lane 11: 35 min ON, lane 12: overnight (~ 8 hours) OFF, lane 13: 2 hours ON, lane 14: 3 hours ON, lane 15:

Molecular Weight Markers; **(B)** lanes 1-4: unrelated products, lane 5: BSA-Br, lane 6: native BSA: lane 7: BSA-PDMAEMA [DMAEMA]/[BSA-Br]=500/1, lane 8: BSA-PDMAEMA [DMAEMA]/[BSA-Br]=1000/1, lane 9: BSA-PDMAEMA [DMAEMA]/[BSA-Br]=1500/1, lane 10: BSA-PDMAEMA [DMAEMA]/[BSA-Br]=2000/1, BSA-Br (I_o), lane 11: β-gal-PDMAEMA, lane 12: β-gal-PAAm, lane 13: β-gal-Br, lane 14: native β-galactosidase, lane 15: Molecular Weight Markers.

Supplementary References

1. Ciampolini, M., Nardi, N. Five-coordinated high-spin complexes of bivalent cobalt, nickel and copper with tris(2-dimethylaminoethyl)amine. *Inorganic Chemistry* **5**, 41−44 (1966). doi:10.1021/ic50035a010

2. Abramoff, M. D., Magelhaes, P. J. Ram, S. J. Image processing with ImageJ. *Biophotonics Int.*, **11**, 36-42 (2004).

3. Yamamichi, S., Jinno, Y., Haraya, N., Oyoshi, T., Tomitori, H., Kashiwagi, K., Yamanaka, M. Separation of proteins using supramolecular gel electrophoresis. *Chem. Commun*. **47**, 10344-10346 (2011). doi:10.1039/c1cc13826j

4. Bolm, C., Dinter, C. L., Seger, A., Hoecker, H., Brozio, J. Synthesis of catalytically active polymers by means of ROMP: An effective approach toward polymeric homogeneously soluble catalysts. *Journal of Organic Chemistry* **64**, 5730−5731 (1999). doi:10.1021/jo990533u

5. Mantovani, G., Lecolley, F., Tao, L., Haddleton, D. M., Clerx, J., Cornelissen, J. J. L. M., Velonia, K. Design and synthesis of *N*-maleimido-functionalized hydrophilic polymers via copper-mediated living radical polymerization: A suitable alternative to PEGylation chemistry. *J. Am. Chem. Soc*. **127**, 2966−2973 (2005). doi:10.1021/ja0430999

6. Le Droumaguet, B., Velonia, K. In situ ATRP-mediated hierarchical formation of giant amphiphile bionanoreactors. *Angew. Chem. Int. Ed.* **47**, 6263−6266 (2008). doi:10.1002/anie.200801007

7. Agut, W., Brûlet, A., Schatz, C., Taton, D., Lecommandoux, S. pH and Temperature responsive polymeric micelles and polymersomes by self-assembly of poly[2-(dimethylamino)ethyl methacrylate]-b-poly(glutamic acid) double hydrophilic block copolymers. *Langmuir* **26**, 10546-10554 (2010). doi:10.1021/la1005693

8. Kowacz, M., Warszyński, P. Beyond esterase-like activity of serum albumin. Histidine-(nitro)phenol radical formation in conversion cascade of p-nitrophenyl acetate and the role of infrared light. *J. Mol. Recognit.* ***32***, e2780 (2019). doi:10.1002/jmr.2780

9. Lecolley, F., Tao, L., Mantovani, G., Durkin, I., Lautru, S., Haddleton, D. M. A new approach to bioconjugates for proteins and peptides ("pegylation") utilising living radical polymerization. *Chem. Commun*., 2026-2027 (2004). doi:10.1039/b407712a

10. Zhang, Q., Li, M., Zhu, C., Nurumbetov, G., Li, Z., Wilson, P., Kempe, K., Haddleton, D. M. Well-defined protein/peptide−polymer conjugates by aqueous Cu-LRP: Synthesis and controlled self-assembly. *J. Am. Chem. Soc*. **137**, 9344−9353 (2015). doi:10.1021/jacs.5b04139

11. Park, Y. K., DeSanti, M. S. S., Pastore, G. M. Production andcharacterization of β-galactosidase from Aspergillus oryzae. *J. FoodSci*.. **44**, 100 (1979). doi:10.1111/j.1365-2621.1979.tb10016.x

12. Dai, H., Li, Y., Zhang, Q., Fua, Y., Li, Y. A colorimetric biosensor based on enzyme catalysis-induced production of inorganic nanoparticles for sensitive detection of glucose in white grape wine. *RSC Adv.*, **8**, 33960-33967 (2018). doi:10.1039/c8ra06347h
